# Charge Regulation Triggers Condensation of Short Oligopeptides to Polyelectrolytes

**DOI:** 10.1021/jacsau.3c00668

**Published:** 2024-03-13

**Authors:** Sebastian P. Pineda, Roman Staňo, Anastasiia Murmiliuk, Pablo M. Blanco, Patricia Montes, Zdeněk Tošner, Ondřej Groborz, Jiří Pánek, Martin Hrubý, Miroslav Štěpánek, Peter Košovan

**Affiliations:** † Department of Physical and Macromolecular Chemistry, Faculty of Science, Charles University, Hlavova 8, Prague 2 128 40, Czech Republic; ‡ Faculty of Physics, University of Vienna, Boltzmanngasse 5, Vienna 1090, Austria; ¶ Vienna Doctoral School in Physics, University of Vienna, Boltzmanngasse 5, Vienna 1090, Austria; § Jülich Centre for Neutron Science JCNS at Heinz Maier-Leibnitz Zentrum (MLZ), Forschungszentrum Jülich GmbH, Lichtenbergstraße 1, Garching 85748, Germany; ∥ Department of Material Science and Physical Chemistry, Research Institute of Theoretical and Computational Chemistry (IQTCUB), University of Barcelona, C/Martí i Franquès 1, Barcelona 08028, Spain; ⊥ Department of Physics, NTNU - Norwegian University of Science and Technology, NO-7491 Trondheim, Norway; # Institute of Macromolecular Chemistry AS CR, Heyrovský square 2, 162 06 Prague 6, Czech Republic

**Keywords:** charge regulation, counterion condensation, polyelectrolyte complexes, electrostatic association, constant pH Monte Carlo, potentiometric titration, p*K*_a_, NMR titration

## Abstract

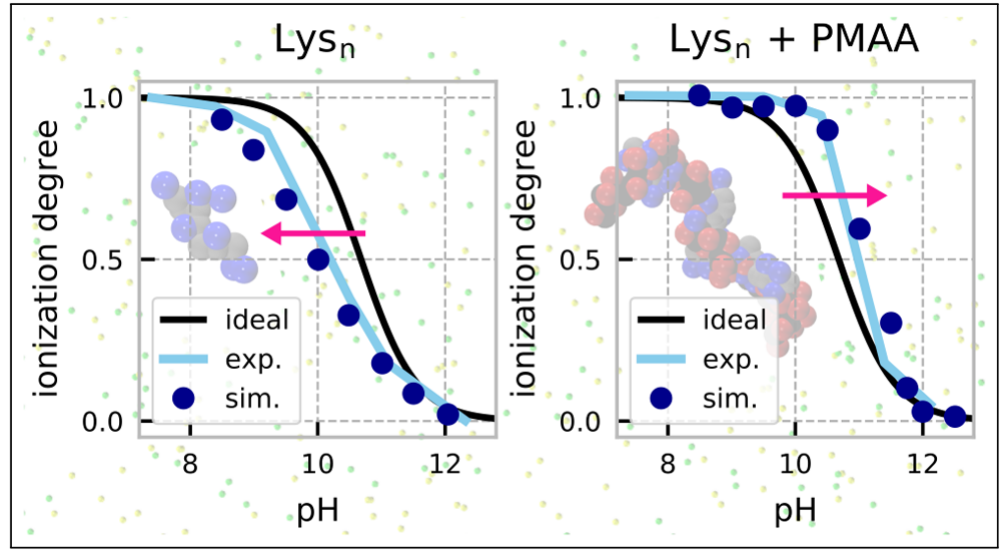

Electrostatic interactions between charged macromolecules are ubiquitous in biological systems, and they are important also in materials design. Attraction between oppositely charged molecules is often interpreted as if the molecules had a fixed charge, which is not affected by their interaction. Less commonly, charge regulation is invoked to interpret such interactions, i.e., a change of the charge state in response to a change of the local environment. Although some theoretical and simulation studies suggest that charge regulation plays an important role in intermolecular interactions, experimental evidence supporting such a view is very scarce. In the current study, we used a model system, composed of a long polyanion interacting with cationic oligolysines, containing up to 8 lysine residues. We showed using both simulations and experiments that while these lysines are only weakly charged in the absence of the polyanion, they charge up and condense on the polycations if the pH is close to the p*K*_a_ of the lysine side chains. We show that the lysines coexist in two distinct populations within the same solution: (1) practically nonionized and free in solution; (2) highly ionized and condensed on the polyanion. Using this model system, we demonstrate under what conditions charge regulation plays a significant role in the interactions of oppositely charged macromolecules and generalize our findings beyond the specific system used here.

## Introduction

Charged moieties are omnipresent in aqueous environments in both the natural and synthetic worlds. From small monatomic ions up to complex molecules with multiple charges, such as proteins, the interactions between such ionic groups are governed mainly by the Coulomb potential, which is nonspecific, noncovalent and long ranged. Electrostatic interactions are vital for living matter; for instance, they provide the driving force for complexation of DNA with positively charged histones,^1,2^ compaction of viral genome,^3^ assembly of actin filaments into bundles by multivalent binders^4^ or formation of complex coacervates,^5,6^ and membraneless organelles.^7^ Ample biopolymers such as RNA, DNA, hyaluronic acid, or many intrinsically disordered proteins bear charged groups,^8−10^ which provide unique means of regulating their charge and organization through changes of pH, type of salt ions or salt concentration. There have been attempts to utilize the above responsiveness in the design of inverse patchy colloids,^11,12^ supramolecular engineering,^13−15^ peptide nanotechnology,^16−18^ or gene delivery.^19,20^ For example, adhesion of highly cationic lysozyme to the anionic bacterial cell wall is important for its bactericidal effect.^21^ Another example is the penetration of SARS-CoV-2 into cells, which is crucially determined by the multivalent charge interaction^22^ of the cationic domains of the spike protein with polyanions on the glycocalyx of the target cells. Accordingly, its entry into the cell can be inhibited by employing polyanions binding to the cationic part of the spike protein or by small oligocations that mask the negative charge of the glycocalyx.^23,24^ Nevertheless, to harvest such applications, we first need a better fundamental understanding of the electrostatic interaction and its mediation by charge regulation, i.e., by a change in the charge state in response to changes in the pH or in other parameters of the local environment.

Although it is generally known that oppositely charged molecules attract each other, it is not easy to tell under what conditions their attraction is strong enough so that they associate. The term counterion condensation has been coined to describe such an interaction in polyelectrolyte solutions. According to the early theory of Manning, counterions should condense on an oppositely charged polyelectrolyte chain until they reduce its effective linear charge density to a threshold value.^25−28^ Notably, in this context, we use the term counterion not only for small monovalent ions but more generally for any oppositely charged ions or molecules present in the solution. The threshold charge density for monovalent counterions amounts to one elementary charge per Bjerrum length. At the threshold charge density, the loss in the translational entropy of counterions upon their condensation is compensated by the approximate enthalpy gain due to electrostatic interactions. The condensed counterions are confined to the vicinity of the polyelectrolyte which is manifested by a lower osmotic pressure of the solution.^29^ The mobility of condensed counterions is significantly reduced because of the confinement; however, they remain sufficiently mobile to dynamically escape from the confinement at the chain. Sometimes, the term counterion condensation is used to describe simple accumulation of counterions near polyelectrolytes by other mechanisms than electrostatic interactions, e.g., due to ion-specific effects, also in situations when the Manning conditions for counterion condensation are not met.^30,31^ Although we acknowledge that these ion-specific effects may be important, in further discussion, we will focus on electrostatic effects, which become dominant if multivalent or oligomeric ions are present in the system.

If multivalent counterions are present in a mixture with monovalent ions, then the multivalent counterions condense first, while the monovalent counterions remain free. This is because a multivalent counterion of valency *z* loses the same amount of translational entropy as a monovalent one, whereas it gains *z*-times more electrostatic energy upon condensation. Therefore, the condensation threshold for *z*-valent counterions can be estimated to be *z*-times lower than that for the monovalent ones. Alternatively, one can argue that if a *z*-valent counterion condenses on a polyelectrolyte, it displaces *z* monovalent counterions, which are released into the solution. The released counterions gain translational entropy *T*Δ*S* = (*z* – 1)*k*_B_*T*, which is the main driving force of the condensation. In contrast, the net change in electrostatic interaction energy is small, resulting in Δ*H* ≈ 0. Therefore, if multivalent counterions are present, then practically all of them condense, displacing the monovalent ones that remain mostly free. Thermodynamic analysis of experimental data,^32,33^ supported by simulations,^34,35^ confirms that the entropy gain due to counterion release^32−34^ and solvent reorganization^35,36^ are the main driving forces of condensation of multivalent counterions, whereas the contribution due to polymer-ion electrostatic interactions seems to be less significant. The condensation of multivalent counterions is used, e.g., for the compaction of DNA by cationic poly(ethyelene imine),^37,38^ spermine or spermidine,^39^ and the same physics can be used to describe the binding of DNA to positively charged histones.^1,2^ The formation of interpolyelectrolyte complexes upon mixing of two oppositely charged polyelectrolytes can be viewed as an extreme case of counterion condensation, also being driven by the release of monovalent ions.^40,41^ For example, the interaction of long polyelectrolytes with short oligomeric counterions could be described as both counterion condensation or polyelectrolyte complexation. However, one would probably describe it as counterion condensation only if the counterions are much smaller than the polymer, and they are present in a much smaller amount. A higher amount of oligomeric counterions would likely result in the precipitation and formation of clusters involving multiple polymer chains. In this case, it would be described as polyelectrolyte complexation. Nonetheless, if the association of two oppositely charged molecules is accompanied by significant conformational changes, such as the compaction of DNA or polyelectrolyte complexation, then not only electrostatics but also other specific interactions may contribute to the net effect.

If weakly acidic or basic groups are involved in the interaction between two oppositely charged molecules, they can undergo charge regulation, i.e., a change in their ionization states as a response to a change in their local environment. The charge regulation in polyelectrolytes composed of identical monomers (polyacids or polybases) decreases their net charge in order to decrease the electrostatic repulsion among like-charged groups. Consequently, charge regulation shifts the effective p*K*_A_ of polyacids to higher values and the effective p*K*_A_ of polybases to lower values than those of the corresponding monomers.^42,43^ Charge regulation in peptides, proteins or synthetic polyampholytes, which contain both acidic and basic groups, may shift the effective p*K*_A_ of these groups in either direction, depending on what kind of charges prevail and how they are distributed in space.^44−51^

Within the mean-field picture, the degree of ionization, α, can be described by the Henderson–Hasselbalch equation, augmented by an additional electrostatic term^52−54^
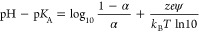
1where *e* is the elementary charge, *z* = ±1 is the valency of the ionized group, and ψ is the local electrostatic potential. In the absence of interactions (ideal gas limit), ψ = 0; therefore, 50% of the groups in the molecules are ionized (α = 0.5) at pH = p*K*_A_. The shift of the effective p*K*_A_ then follows as p*K*_A_^eff^ = p*K*_A_ + (*zeψ*)/(*k*_B_*T* ln10).^52^ In our previous studies,^54−59^ the above mechanism of shifting the effective p*K*_A_ has been termed the *polyelectrolyte effect*. The latter has been contrasted with the *Donnan effect*, caused by uneven partitioning of H^+^ ions between different phases in two-phase systems, resulting in different pH values in these two phases. A similar approach can be applied at the nanoscale, if the system can be divided into two parts which are both approximately electroneutral, as it has been done in theories of polyelectrolyte brushes and star polyelectrolytes.^58,60^ In the current context, we are dealing with a single phase that is homogeneous on the macroscopic scale. Although it may be considered heterogeneous at the nanoscale, it cannot be divided into two parts, which could be considered to be approximately electroneutral. Consequently, the Donnan effect is not applicable in the current context, and all changes in the ionization response can be attributed to intermolecular interactions (polyelectrolyte effect).

Several theoretical studies have suggested that charge regulation may significantly contribute to interactions between two oppositely charged macromolecules. Rathee et al.^61^ predicted by simulations that if a polyacid and polybase interact while the solution pH is close to the p*K*_A_ of one of them, then that molecule charges up to make the attraction more favorable. They termed this effect “strong associative charging”, in contrast to “weak associative charging”, which occurs if the pH is close to the p*K*_A_ of both of these molecules, so that they both simultaneously charge up upon interaction. In accordance with that, Staňo et al.^56^ predicted by simulations that charge regulation may enable the formation of electrostatically cross-linked gels at a pH value when one of the interacting macromolecules would be uncharged in the absence of its oppositely charged interaction partner. This theoretical prediction seems to explain why some experiments^62,63^ observed complexation of charge-regulating polyelectrolytes at pH values when these polyelectrolytes should not yet be sufficiently charged. The same mechanism could explain the binding of short DNA to supramolecular polymers containing mobile cationic moieties.^64^ Along these lines, simulations by Staňo suggested that short oligopeptides could increase their net charge when interacting with oppositely charged polyelectrolytes, such as DNA.^65^ Subsequently, Lunkad et al.^66^ have shown that if peptides interact with polyelectrolytes, then charge regulation may allow the peptides to switch the sign of their net charge, provided that p*K*_A_ values of acidic or basic groups on the peptide are close to the solution pH. The simulation study by Lunkad et al.^66^ provided an alternative explanation of why proteins adsorb on polyelectrolytes on the “wrong” side of the isoelectric point, i.e., at pH values when the net charge of the protein has the same sign as the polyelectrolyte. Previously, this phenomenon has been explained by the release of counterions, enabled by patchy charge distribution on the protein.^67^ Interestingly, the effect of charge regulation could be interpreted so that it facilitates the formation of oppositely charged patches, thus enabling the attraction of the protein to the oppositely charged macromolecule, accompanied by the release of monovalent counterions. Therefore, although most experiments can be quantitatively described by counterion release, this does not exclude the possibility that charge regulation plays an important role. Additionally, the important role of charge regulation has been demonstrated in studies of phase-separating systems forming interpolyelectrolyte complexes or polyelectrolyte multilayers. In these systems, highly ionized and weakly ionized forms of the same molecule have been found to coexist at equilibrium in two different phases, which provide different microenvironments for the charge-regulating species.^68−73^ More specifically, these studies have shown that ionization is enhanced in the polyelectrolyte phase. In our current study, we show that charge regulation can enable the simultaneous coexistence of these two ionized forms within a single homogeneous phase.

**Figure 1:**
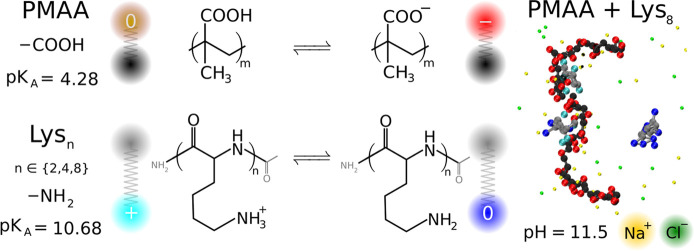
Overview of the investigated system: long anionic poly(methacrylic acid) (PMAA) and short cationic oligolysines (Lys_*n*_). The chemical structures on the left show the different ionization states. The schematic next to each chemical structure represents the coarse-grained model that we used for the corresponding chain. Color code: gray and black = backbone groups; orange = nonionized acidic groups; red = ionized acidic groups; cyan = nonionized basic groups; blue = ionized basic groups; green = small anion; yellow = small cation.

To demonstrate the significant role of charge regulation, we designed a simple model system, consisting of a long polyanion, which has a constant charge in the relevant pH range, and short oligocations, which can regulate their charge. Our model polyanion is poly(methacrylic acid), (PMAA, monomer p*K*_A_ ≈ 4.28)^42^ and the polycations are oligolysines composed of 2, 4, and 8 lysine residues (ϵ-amino group of the monomer p*K*_A_ = 10.68),^74^ as illustrated in Figure 1. The molar ratio of lysine to methacrylic monomeric units was 1:2, the same as in the experiments. The excess of methacrylic monomers should ensure that even if all lysines condense on the PMAA chains, there is still enough negative charge left on the PMAA to keep the polymer stretched and soluble. Protective groups at the C-end and N-end of each oligolysine ensured that the ionization response was not affected by free carboxyl and amino groups. Using these model molecules, we studied the ionization of oligolysines in aqueous solutions with and without PMAA, demonstrating significant differences between the two. In either case, both oligolysines and PMAA were present at relatively low concentrations in excess NaCl, which ensured a fixed ionic strength irrespective of the pH. The pH was adjusted to the desired value by adding extra NaOH or HCl. Full details on the studied system, simulations, and experiments are provided in the Methods section and in the Supporting Information (SI).

Based on the considerations described above, a significant charge regulation may be expected if the solution pH is close to p*K*_A_ = 10.68 for the lysine ionizable groups. In the following, we demonstrate using both simulations and experiments, that if the oligolysine polycations are sufficiently long, then they are attracted to the PMAA polyanion at pH values at which the lysines should be uncharged in the absence of the polyanion. At slightly higher pH values, this attraction vanishes, confirming that it is indeed triggered by charge regulation and not by other interactions, such as the hydrogen bonding or hydrophobicity. By comparing oligolysines of various chain lengths, we can further show that indeed electrostatic repulsion between like-charged groups on the lysines suppresses their ionization and shifts their effective p*K*_A_ to lower values, as expected for polybases, and this effect becomes stronger as the chain length increases. On the contrary, their interaction with oppositely charged PMAA completely reverses this trend, enhancing the ionization and shifting the effective p*K*_A_ of oligolysines in the opposite direction. As the lysine chain length is increased, the transition between the nonionized and fully ionized state becomes more abrupt, in accordance with the strong associative charging numerically predicted by Rathee et al.^61^ In our simulations and experiments, we demonstrate that if the oligomeric counterion is long enough, then it may coexists in two different states within the same solution: (1) highly charged, condensed on the polyelectrolyte; (2) almost uncharged, free in solution.

## Results and Discussion

**Figure 2:**
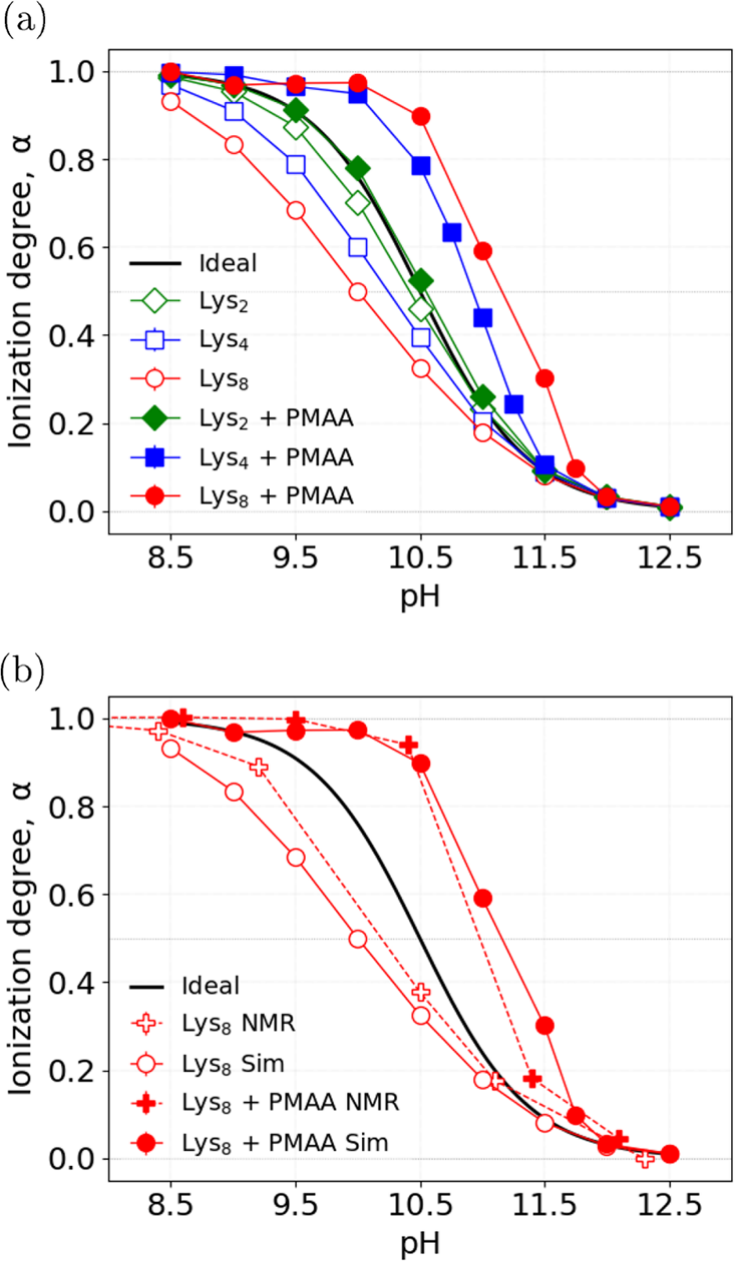
Degree of ionization of Lys_n_ with *n* ∈{2, 4, 8} as a function of pH. (a) Simulation results for Lys_*n*_ in the absence of PMAA (empty symbols) and Lys_*n*_ in the presence of PMAA (filled symbols). (b) Simulations (circles) and NMR results (crosses) for Lys_8_ in the absence of PMAA (empty symbols) and Lys_8_ in presence of PMAA (filled symbols).

Before discussing the interactions between oligolysines and PMAA, it is instructive to discuss the behavior of oligolysines in solution, in the absence of PMAA. Figure 2 shows that the ionization curves of oligolysines are shifted toward lower pH values, as compared to the ideal Henderson–Hasselbalch result, obtained using eq 1 with ψ = 0. This shift increases as the chain length of the oligolysine is increased, reflecting an increase in the electrostatic repulsion between like-charged groups in the oligolysines. The electrostatic repulsion implies an additional free energy cost of the ionization, which causes a diminution in the ionization degree. This shift can be quantified by the effective p*K*_A_ value of the side-chains of oligolysines being lower than the corresponding p*K*_A_ of the lysine monomer. In addition to the shift in the effective p*K*_A_ value, the curves become less steep as the chain length of lysines is increased, in line with previous studies on the titration of various polyacids and polybases in solution.^42,53,75−79^

Figure 2 also shows that the shifts of the ionization curves of oligolysines may be reversed if these lysines interact with oppositely charged PMAA. In the following, we demonstrate that this enhancement of ionization is caused by electrostatic attraction between the cationic oligolysines and the anionic PMAA. This attraction counterbalances the repulsion between like charges within the lysine molecules, shifting the ionization curves in Figure 2 toward higher pH values (higher effective p*K*_A_). Interestingly, this effect causes the ionization degree of Lys_2_ interacting with PMAA to almost perfectly match the ideal Henderson–Hasselbalch result. Nevertheless, this apparent ideality is caused by mutual compensation of two nonideal effects. For longer lysines, composed of 4 or 8 units, the ionization curves in Figure 2 are shifted further to the right of the ideal curve, suggesting that the intramolecular repulsion among the like charges on longer lysines is overcompensated by interaction with the oppositely charged PMAA. In addition, as the chain length of lysines is increased, the ionization curves in the presence of PMAA become increasingly steep, opposite to what we observed for lysines in the absence of PMAA. By extrapolating this observation to longer chains, one can hypothesize that the transition should approach an infinitely steep first-order transition. In such case, a simultaneous coexistence of fully ionized and nonionized lysines should be observed within the same system. Later, we show that such coexistence is indeed observed in our simulations. Figure 2b shows that the ionization degree of Lys_8_, experimentally determined from NMR spectra, agrees well with our simulations, both in the presence and in the absence of PMAA. Thus, our simulations and experiments confirmed that the interaction with oppositely charged polyelectrolytes can reverse the known effect of electrostatic interactions on the p*K*_A_ shift of polyelectrolytes and that the extent of this reversal can be tuned by the chain length.

**Figure 3:**
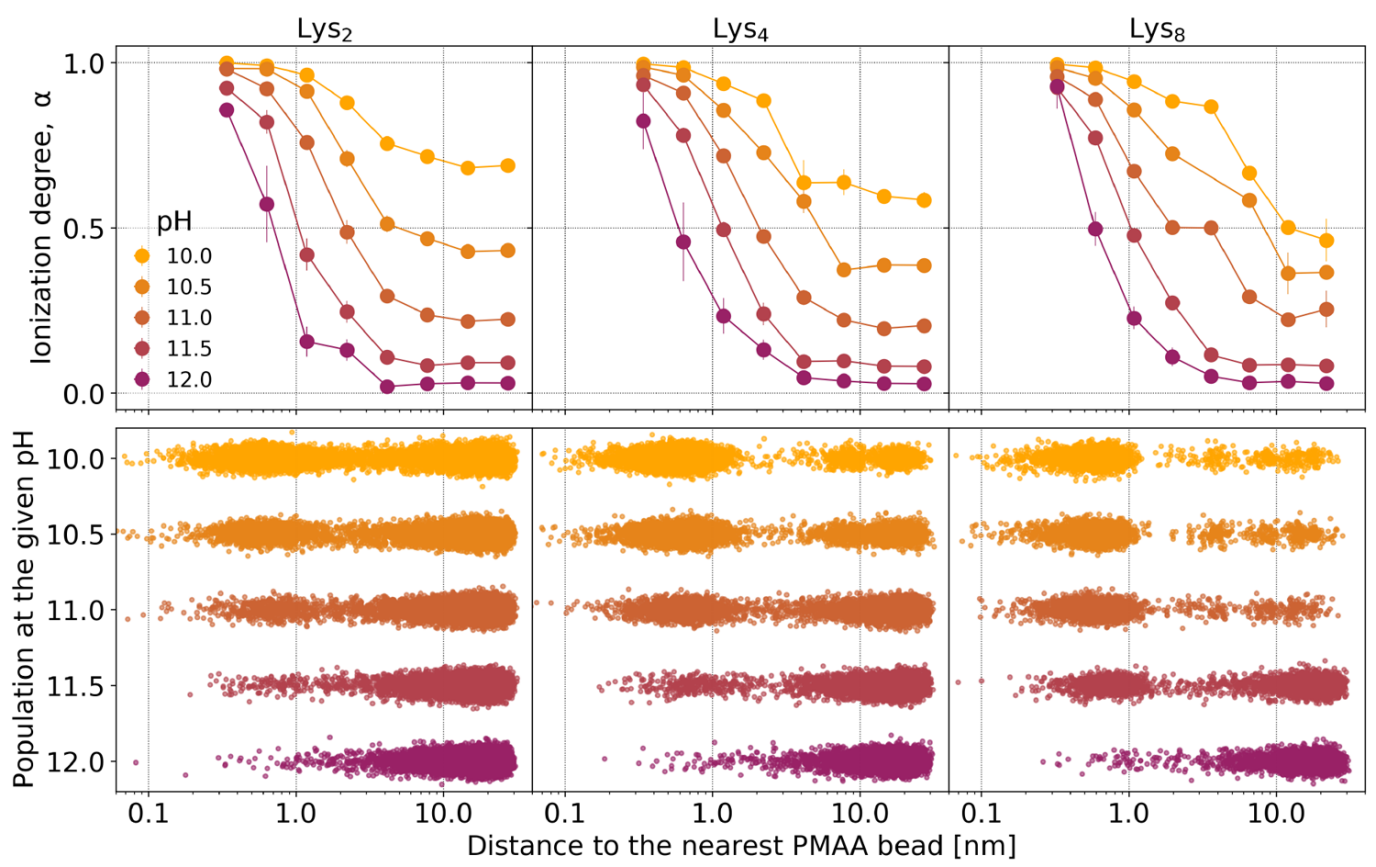
Simulation results for the ionization degree of Lys_n_ (top row) and the relative population of lysines (bottom row) as a function of their distance to the nearest bead of the PMAA. Each point in the bottom panel corresponds to the distance between the center of mass of one oligolysine molecule and the closest PMAA bead in each of the simulation frames.

The anticipated coexistence of lysines in two different states is evidenced in Figure 3 which shows the populations of oligolysines as a function of their distance from the nearest PMAA monomer, obtained from simulations. These plots show data aggregated over all simulation frames, such that one data point represents one oligolysine observed at a particular distance from the PMMA. To better visualize the populations, a Gaussian scatter has been applied to these points in the vertical direction. In most panels, we observed two clusters of data points: one within about 1 nm, and another one beyond 10 nm. Notably, first cluster of points is at a distance comparable to the size of a small ion and much smaller than the size of PMAA chain, therefore, it corresponds to condensed oligolysines. On the contrary, the second cluster is at a distance greater than the size of PMAA, therefore, it corresponds to free lysines. As an alternative representation, we plot the same data aggregated into a histogram in Figure S1. As evidenced by the plots of ionization degree as a function of distance in Figure 3, the condensed oligolysines are highly ionized, whereas those far from the polyelectrolyte are much less ionized. This local variation of the degree of ionization of lysines well correlates with the local variation in the local concentration of H^+^ ions as a function of distance from the PMAA monomers, shown in Figure S2. Indeed, we observe an increase in the local concentration of H^+^ ions near the PMAA chain, which could alternatively explain the increase in the degree of ionization of Lysine. The local concentration of H^+^ ions, sometimes incorrectly termed the “local pH”,^52,78^ reflects the local variation of the electrostatic potential, which determines the excess contribution to the free energy of dissociation of the titratable groups on oligolysines. Figure S2 demonstrates that the “local pH” at the PMAA chain is slightly lower for Lys_2_ than Lys_4_ and Lys_8_, which correlates with slightly lower ionization of Lys_2_ at the PMAA chain. On the other hand, at pH = 12, the “local pH” is around 10.5 or higher, suggesting that the lysines should not be fully ionized when condensed on the PMAA chain(cf. Figure 2). However, Figure 3 shows that, even at the highest pH values, all lysines are almost fully ionized when condensed on the PMAA, indicating an additional contribution that cannot be explained solely by the “local pH” effects.

**Figure 4:**
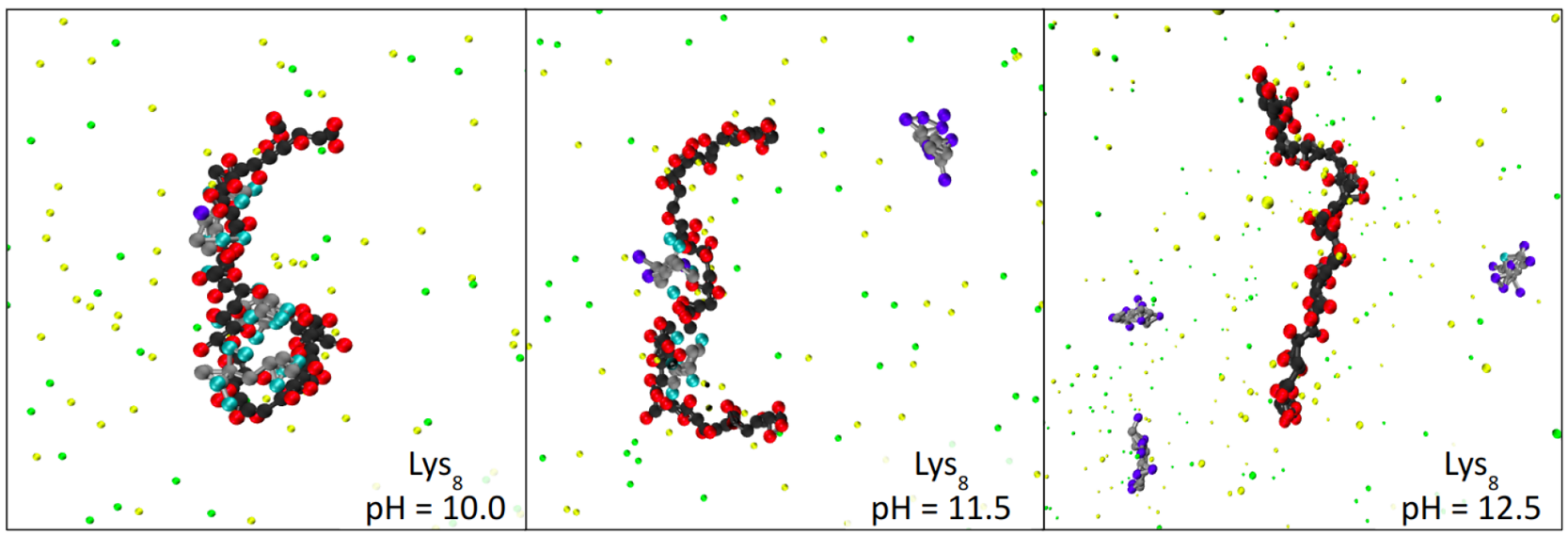
Simulation snapshots of the Lys_8_ interacting with PMAA. At pH = 10.0 (≲ p*K*_A_^eff^) the lysines are highly ionized and condensed on the PMAA chain, and at pH = 12.5 (≳ p*K*_A_^eff^) the lysines are weakly ionized and free in solution. However, at pH = 11.5 (≈ p*K*_A_^eff^), the two different ionization states coexist in the solution. Color code is the same as that in Figure 1.

As the pH is increased, the number of highly ionized lysines close to the polyelectrolyte decreases and consistently the number of weakly ionized lysines far from the polyelectrolyte increases. For Lys_2_, we observe that the population of highly ionized lysines significantly decreases at pH > 10.5, whereas for longer lysines this population persists up to much higher pH values. Furthermore, the populations of highly charged and uncharged lysines are much more clearly separated for longer lysine chains, whereas the shorter lysines are more likely to be found at intermediate distances and intermediate ionization degrees. Within the counterion condensation framework, this effect could be interpreted so that the highly ionized lysines condense on the PMAA chain, whereas the weakly ionized ones do not condense. The same effect could be interpreted within the charge regulation framework so that the lysines increase their charge to enable their condensation on the chain. In practice, both charge regulation and condensation occur simultaneously, causing that longer lysines exist within the same system in two distinct states: either highly charged and condensed on the polyelectrolyte or weakly charged and free in solution (noncondensed) as can be observed in the simulation snapshots of the system with Lys_8_ in Figure 4. Analogous simulation snapshots of the systems with Lys_2_ and Lys_4_ are shown in Figure S5.

**Figure 5:**
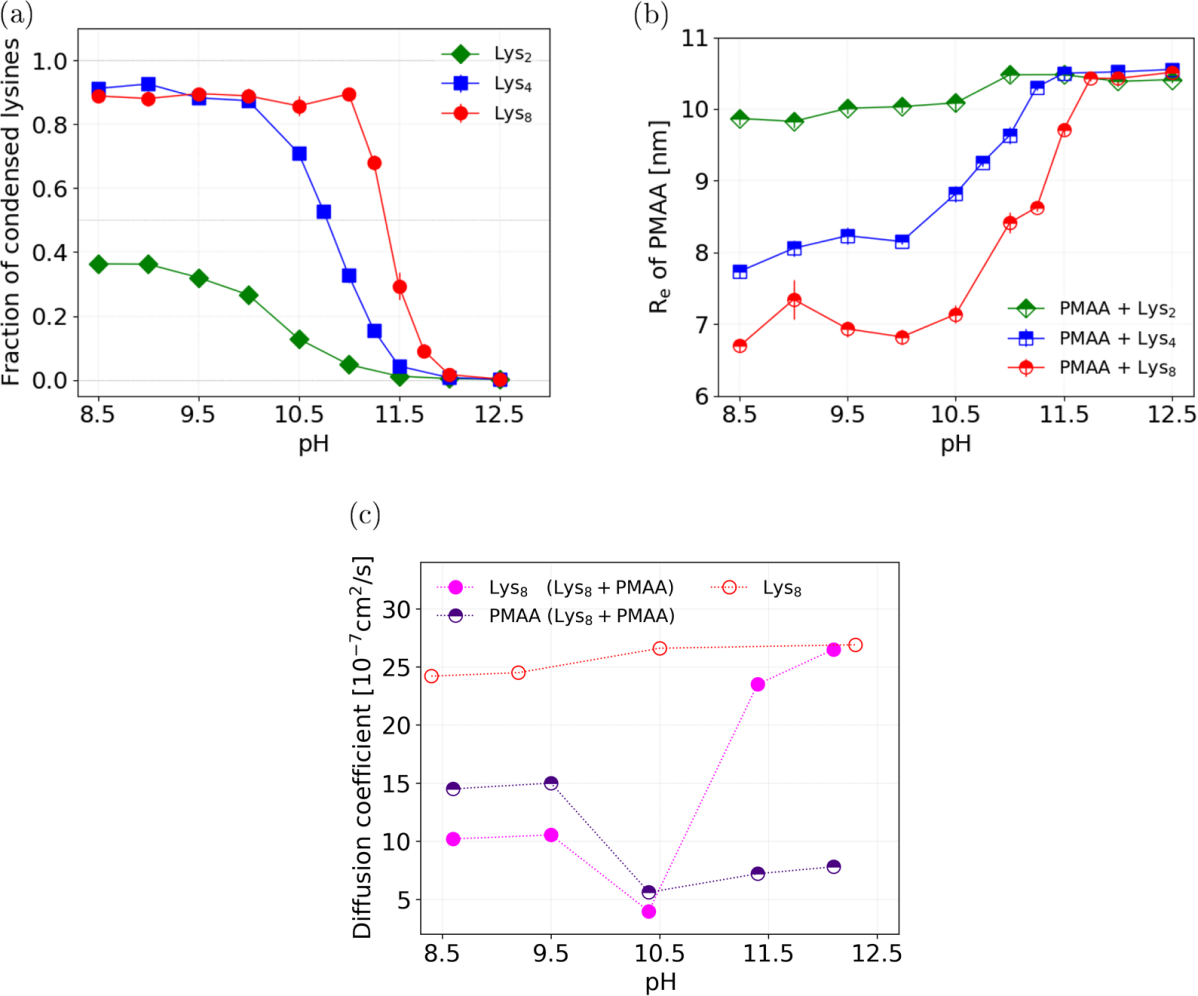
Condensation, swelling of the PMAA for Lys_*n*_ with *n* ∈{2, 4, 8} and diffusion of Lys_8_ in the presence and absence of PMAA as a function of pH. (a) Fraction of Lys_*n*_ condensed on the PMAA, obtained from simulations. (b) End-to-end distance of PMAA in the presence of Lys_*n*_, obtained from simulations. (c) Diffusion coefficient of Lys_8_ and PMAA in a common solution (labeled as Lys_8_ + PMAA), compared to Lys_8_ in the absence of PMAA, determined from DOSY NMR experiments.

To quantify the condensation of lysines on the PMAA chain, we computed the fraction of free lysines at various pH values, shown in Figure 5. We considered the lysines to be condensed if they were closer to the PMAA chain than 2 nm. We chose this threshold because it approximately corresponds to the local minimum in the populations of lysines as a function of distance to the nearest PMAA bead, shown in Figure 3. Figure 5a shows that at pH ≲ 10 about 30–40% of the shorter lysines (Lys_2_) condense on the PMAA chain, whereas the remaining 60–70% are free in solution. In contrast, about 90% of the longer lysines (Lys_4_ and Lys_8_) condense on PMAA under the same conditions. This is expected because the longer lysines bear a higher charge when fully ionized; therefore, they condense more strongly. As the pH is increased, the fraction of condensed Lys_2_ gradually decreases and approaches zero at pH ≈ 11.5. The fraction of condensed Lys_4_ remains high up to a higher pH and then decreases more abruptly, as compared to Lys_2_. Finally, the Lys_8_ exhibits a rather sharp transition between completely condensed and completely free states within about 0.5 units of pH, resembling a first-order phase transition.

The condensation of lysines at various pH values is further reflected by changes in the PMAA conformation, as evidenced by the plot of its end-to-end distance as a function of pH in Figure 5b. In all cases, the condensed lysines cause shrinkage of the PMAA chain. As the pH is increased, the end-to-end distance of PMAA increases, reaching the same saturation value at a high pH, as the lysines gradually lose their charge and detach from the PMAA chain. Therefore, this increase in end-to-end distance very well correlates with the decrease of the fraction of condensed lysines (Figure 5a) and concomitantly it correlates with the decrease in their degree of ionization (Figure 2). The condensation of Lys_2_ has a much smaller effect on the end-to-end distance of PMAA than the condensation of Lys_4_ or Lys_8_. The difference between Lys_2_ and Lys_4_ could be explained by the lower fraction of condensed Lys_2_. However, the same argument cannot explain the difference between Lys_4_ and Lys_8_ at low pH because they are both fully ionized and condense to the same extent at pH ≲ 10. Therefore, the difference in the end-to-end distance of PMAA interacting with Lys_4_ and Lys_8_ demonstrates that there is an additional cooperativity between the ionization and condensation for longer lysines, whereas this cooperativity is very weak for short lysines.

Finally, Figure 5c shows the diffusion coefficients of Lys_8_ and PMAA, determined from DOSY NMR measurements at various pH values. Expectedly, the diffusion coefficient of Lys_8_ in solution in the absence of PMAA is only weakly affected by the pH. On the contrary, the diffusion coefficient of Lys_8_ in the presence of PMAA strongly depends on the pH. At low pH, when the lysine is charged and condensed on the PMAA chain, the diffusion coefficients of Lys_8_ and PMAA are very similar and significantly lower than the diffusion coefficient of lysine without PMAA. As the pH is increased, the diffusion coefficient of Lys_8_ abruptly increases, attaining the same value as the diffusion coefficient of lysine in the solution without PMAA. An independent piece of evidence of the interaction between PMAA and Lysines is provided by the cross peak between PMAA and Lys_8_ in NOESY NMR spectra, shown in Figure S15. This peak is present at pH ≤ 10.4, indicating that PMAA and Lys_8_ interact at lower pH values, but it vanishes at pH ≥ 11.4, indicating that they no longer interact at the higher pH values. Therefore, we can conclude that not only simulations but also experiments indicate that the long lysines condense on the PMAA chain at pH ≲ 10.4, whereas they remain free at pH ≳ 11.4.

## Conclusions

Using a model system composed of long anionic poly(methacrylic acid) (PMAA) and short polycationic oligolysines, we demonstrated how charge regulation affects interactions between two oppositely charged macromolecules. From both experiments and simulations we observed that the net charge of oligolysines in the absence of PMAA is lower than that of the parent monomer, which can be quantified by a shift in their effective p*K*_A_ values. This shift is stronger for longer oligolysines in accordance with the established knowledge in the field of polyelectrolytes. However, if these oligolysines interact with the anionic PMAA, then they condense on the polyanion. This condensation is accompanied by an increase in the net charge of the oligolysines. Ultimately, this increase in the ionization reverses the p*K*_A_ shifts of lysines, causing that the effective p*K*_A_ is higher than that of the parent monomer. The latter effect is enhanced as the length of the lysines is increased. Furthermore, our simulations have shown that the longer oligolysines simultaneously exist in two different ionization states within the same system: one highly ionized and condensed and another one practically nonionized and free in solution. Notably, individual lysine oligomers are dynamically exchanged between the condensed and free states. The correlation between condensation and ionization was further confirmed by our NMR experiments. The transition between these two states as a function of pH becomes sharper as the chain length of oligolysine is increased, resembling a first-order transition. If we extrapolate our findings to longer chains, they suggest that charge regulation should play a significant role in interactions between oppositely charged macromolecules, enhancing their ionization and thereby enabling association at pH values where one or both macromolecules should be uncharged in the absence of the oppositely charged polymeric partner. This effect should be particularly important if the pH is not far from the p*K*_A_ of one or both macromolecules. The effect is not unique to peptides interacting with polyelectrolytes but should apply to any oligomeric counterions if the solution pH is close to the p*K*_A_ value.

The simplicity of both the experimental setup and the coarse-grained simulation model underscores the universality of our results. The prospect of tailoring the complexation by engineering the charge regulation is relevant mainly for materials design and biomedical applications. For example, complexation of small cationic pro-inflammatory cytokines, such as cathelicidin, with polyanions, such as extracellular DNA, is crucial for the defense of the organism against bacteria^80^ and also for development of autoimmune diseases.^81^ Likewise, anionic polysaccharide heparin, clinically used as a coagulant, can be neutralized with positively charged peptide protamin.^82,83^ The heparine-protamin complexation can be observed also at pH ≈ 7.4 corresponding to the blood conditions, where protamin should be only weakly charged. At the same time, low molecular weight (fractionated) heparin exhibits weaker complexation with protamin,^84^ which can be explained by the mechanisms described by our study. The fundamental understanding of the interplay of oligocation length and charge regulation presented above can guide design of heparin sensors^85^ or development of alternative antidotes. Similarly, the mechanisms revealed in our study can explain why gels fromed by charged cellulose nanocrystals and poly(allylamine) remain ionized and stable over a broader pH range than could be expected from their solution behavior.^86^ The extrapolation of our results from short peptides and charged oligomers also to complex molecules, such as proteins containing lysine-rich or carboxylate-rich sequences, is in principle possible. Nevertheless, it is known that in such complex environments hydrogen bonding^87−89^ and other interactions can become as important as electrostatics. For such systems, quantitative simulations with predictive power would require refinement of our models by including the hydrogen bonds, as is currently underway.

## Methods

### Simulation Model and Method

To gain detailed insights into charge regulation in the PMAA-lysine system, we employed computer simulations using a coarse-grained bead–spring model in an implicit solvent with explicit ions. In our model, each monomeric unit was represented by two spherical beads, one representing the polymer backbone and the other one representing the side chain, as illustrated in Figure 1. Parameters of this model were set using semiempirical estimates based our previous studies.^44,66,90,91^

The PMAA consisted of 48 monomeric units and the oligolysines consisted of *n* ∈{2, 4, 8} units, denoted as Lys_n_. The number of lysine oligomers was chosen such that the molar ratio of lysine monomeric units to PMAA was 1:2, the same as in the experiments. The *C*-end and the *N*-end were not charged in our model of oligolysines because it was designed to represent the oligopeptides used in our experiments, which had both ends protected by nonionizable groups. In addition to PMAA and oligolysines, small ions (Na^+^, Cl^–^) were present in the system to ensure ionic strength *I* = 0.01 M. We assumed that PMAA side chains were fully ionized because we were interested only in pH > 8 which is much greater than p*K*_A_^PMAA^, so PMAA should be fully ionized. By contrast, the ionization states of lysine side chains were allowed to fluctuate.

The chain length of PMAA in simulations, *m* = 48, was chosen such as to make it much longer than all lysine oligomers yet not too long to enable efficient sampling of the configuration space in simulations. Our previous studies of similar models suggested that the ionization and local conformational properties of polyelectrolytes do not change much at chain lengths *m* ≳ 50.^78^ We chose *m* = 48 because it is divisible by *n* ∈{2, 4, 8}, so that all simulations could be performed at 1:2 molar ratio of lysine to methacrylate monomeric units. Based on the above considerations, simulated PMAA chains were much shorter than *m* ≈ 1150 used in our experiments; this difference should not significantly affect our observations and conclusions. To support this claim, we ran a set of simulations of PMAA with *m* = 96 and Lys_n_ at selected pH values at the same molar ratio and concentration as our original simulations. These simulations, provided in SI, section 1.5, show that doubling of the PMAA chain length has no significant effect on the interpretation of the results.

The molar ratio of lysine to methacrylic units 1:2 was chosen based on earlier simulations performed by Roman Staňo.^65^ In this thesis, Staňo showed that at higher molar ratios the oligopeptides condense on the polyanion to such an extent that they almost fully compensate its charge. This causes significant compaction of the chain, which should cause precipitation in an experimental system. The precipitation is undesired because the polymer and peptide contents in the precipitate might be different from those of the bulk solution. Simultaneously, a high peptide content is desired to ensure favorable signal-to-noise ratio when studying its behavior both in simulations and experiments. Thus, the 1:2 lysine to methacrylate molar ratio was chosen as a compromise between these two competing requirements.

We sampled the ionization states of lysine side chains using the constant-pH method,^92^ with p*K*_A_^lys^ = 10.68 as the input parameter. This method entails a Monte Carlo (MC) procedure, in which the ionization state is changed from protonated to deprotonated or vice versa, while simultaneously inserting or deleting a counterion in order to keep the simulation box electroneutral, represented by a schematic chemical reaction
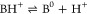
2The acceptance probability of the MC trial move is given by^92^
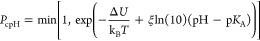
3where Δ*U* is the change in potential energy, ξ = 1 if the base group is being deprotonated, and ξ = −1 if it is being deprotonated in the reaction given by eq 2. The MC moves for sampling the ionization states were coupled to sampling of the configuration space by Langevin Dynamics. The degree of ionization was then computed as an ensemble average over the ionization states in different configurations sampled during the simulation. All simulations were performed using the software ESPResSo v4.1.4.^93,94^ Full details on the simulation model, simulation protocol, and data processing are provided in the SI.

### Experimental Section

To complement the simulations, we studied experimentally the ionization of oligolysines and their interactions with poly(methacrylic acid) using potentiometric titrations and nuclear magnetic resonance (NMR). The simulated PMAA chains consisted of *m* ≈ 1150 monomeric units estimated from the average molar mass. The Lys_*n*_ samples with *n* ∈{2, 4, 8} were custom-synthesized at high purity. The *C-* end of each oligolysine was protected by a primary amide −CONH_2_, and the *N-* end was protected by an acetamido group −NHCOCH_3_ in order to ensure that the ionization response was not affected by free carboxyl and amino groups. From the potentiometric titrations, we determined the net charge of oligolysines as a function of the pH, which enabled us to validate the simulation model. From NMR chemical shifts, we determined the degree of ionization of oligolysines in solutions with and without PMAA, demonstrating significant differences between the two. Additionally, we used NMR to determine the diffusion coefficients of PMAA and oligolysines, which allowed us to determine if the two molecules diffuse independently or not. Full details on the experiments and data analysis are provided in the SI.

## Supporting Information Available

Experimental procedures, details on simulation methods, and additional results (PDF)
