# Supplementary material for: Charge Regulation Triggers Condensation of Short Oligopeptides to Polyelectrolytes
Source: JACS Au. 2024 Mar 13;4(5):1775–85. doi: 10.1021/jacsau.3c00668 (PMC11134362; doi:10.1021/jacsau.3c00668)
Supplement: Supplementary file 1 — au3c00668_si_001.pdf [file au3c00668_si_001.pdf]

Supporting Information: Charge regulation triggers condensation of short oligopeptides to polyelectrolytes

Sebastian P. Pineda,[†] Roman Staňo,[‡,¶] Anastasiia Murmiliuk,[§] Pablo M. Blanco,[‖,†,⊥] Patricia Montes,[†] Zdeněk Tošner,[†] Ondřej Groborz,[#] Jiří Pánek,[#] Martin Hrubý,[#] Miroslav Štěpánek,[*,†] and Peter Košovan[*,†]

†*Department of Physical and Macromolecular Chemistry, Faculty of Science, Charles University, Hlavova 8, 128 40 Prague 2, Czech Republic*

‡*Faculty of Physics, University of Vienna, Boltzmanngasse 5, 1090 Vienna, Austria*

¶*Vienna Doctoral School in Physics, University of Vienna, Boltzmanngasse 5, 1090 Vienna, Austria*

§*Jülich Centre for Neutron Science JCNS at Heinz Maier-Leibnitz Zentrum (MLZ), Forschungszentrum Jülich GmbH, Lichtenbergstraße 1, 85748 Garching, Germany*

‖*Department of Material Science and Physical Chemistry, Research Institute of Theoretical and Computational Chemistry (IQTCUB), University of Barcelona, C/Martí i Franquès 1, 08028 Barcelona, Spain*

⊥*Department of Physics, NTNU - Norwegian University of Science and Technology, NO-7491 Trondheim, Norway*

#*Institute of Macromolecular Chemistry AS CR, Heyrovský square 2, 162 06 Prague 6, Czech Republic*

E-mail: miroslav.stepanek@natur.cuni.cz; peter.kosovan@natur.cuni.cz

Contents

1 Simulations **S-3**

1.1 Simulation model S-3

1.2 Simulation method and protocol S-5

1.3 The distribution of lysines and H^+ ions around PMAA S-7

1.4 Simulation snapshots S-11

1.5 The effect of the PMAA chain length S-11

2 Experimental **S-13**

2.1 Materials S-13

2.2 Potentiometric titration S-13

2.2.1 Ionization response from potentiometric titrations S-14

2.2.2 Titration of free lysines S-15

2.3 Nuclear magnetic resonance spectroscopy S-16

2.3.1 Preparation of samples S-16

2.3.2 NMR measurements S-17

2.3.3 Ionization response from NMR S-18

2.3.4 13C NMR spectra of Lys_8 and PMAA + Lys_8 at various pH values . S-18

2.3.5 1 H NMR spectra of ${Lys_8}^+$ and Lys_8 + PMAA S-25

2.3.6 Analysis of DOSY spectra of pure Lys_8 . . . . S-28

2.3.7 Analysis of DOSY spectra of PMAA + Lys_8 . . . . S-30

2.3.8 NOESY spectra S-33

References **S-36**

1 Simulations

1.1 Simulation model

We used bead-spring coarse-grained models for both PMAA and oligolysines. Each monomeric unit was represented by two spherical beads, one of which represented the backbone and the other one the ionizable side chain. The PMAA chain consisted of 48 monomeric units and the oligolysines consisted of $n \in \{2,4,8\}$ lysine units. In addition, salt ion pairs Na^+Cl^- were added to the system to achieve the desired ionic strength. We initialize the system with fully charged oligolysines, adding additional Cl^- counterions to compensate the charge of the oligolysines.

We sampled the ionization equilibrium of lysine side chains, following the schematic acid-base reaction

$$\mathrm{BH^+} \rightleftharpoons \mathrm{B} + \mathrm{X^+} \tag{1}$$

where B stands for the basic group on the lysine side chain with $\mathrm{p}K_\mathrm{A} = 10.68$, which we set based on Ref.[S1] The symbol X^+ stands for a generic coion that is inserted into the simulation box to maintain electroneutrality of the system. The switching between protonated and deprotonated states of the lysine side chains was performed using Monte Carlo titration moves in the constant pH ensemble, as detailed in the following subsection. Because we simulated only systems at basic pH values, such that the pH was much greater than the $\mathrm{p}K_\mathrm{A} \approx 4.5$ of the PMAA, we considered the PMAA side chains to be permanently ionized.

The simulations were carried out with one chain of PMAA in the simulation box. The number of oligolysines was chosen such that the molar ratio of lysine to methacrylic monomeric units was 0.5, *i.e.*, $N_\mathrm{c} = 12$ chains of Lys_2, $N_\mathrm{c} = 6$ chains of Lys_4 or $N_\mathrm{c} = 3$ chains of Lys_8. The simulation box length $L_\mathrm{box} = 43.7\,\mathrm{nm}$, was chosen such that the concentration of methacrylic units was $c_\mathrm{PMAA} = 1.0\,\mathrm{mM}$. The salt concentration of $c_\mathrm{salt} = 10.0\,\mathrm{mM}$ was achieved by adding 480 Na^+Cl^- ion pairs. The simulations of free oligolysines in solution were performed in an analogous manner with the concentration of lysine units fixed at

$c_{\rm lys} = 1.0\,{\rm mM}$.

The steric repulsion between all beads was represented using Weeks-Chandler-Andersen potential (WCA),

$$U_{\rm WCA}(r) = \begin{cases} 4\epsilon \left[\left(\frac{\sigma}{r}\right)^{12} - \left(\frac{\sigma}{r}\right)^{6} + \frac{1}{4} \right] & r \leq 2^{1/6}\sigma \\ 0 & r > 2^{1/6}\sigma \end{cases} \tag{2}$$

where we used $\epsilon = 1\,k_{\rm B}T$ and $\sigma = 0.35\,{\rm nm}$, which defined the effective particle size.

The monomeric units were bonded using a harmonic potential given by

$$U_{\rm bond}(r) = \frac{1}{2} k_{\rm h} (r - r_0)^2 \tag{3}$$

with an arbitrary stiffness constant $k_{\rm h} = 50 k_{\rm B}T\,{\rm nm}^{-2}$ for all bonds. The equilibrium lengths were chosen as $r_0 = 0.150\,{\rm nm}$ for the backbone of the PMAA, $r_0 = 0.150\,{\rm nm}$ for the side-chain of the PMAA, based on Ref.[S2] $r_0 = 0.382\,{\rm nm}$ for the backbone of the oligolysines and $r_0 = 0.558\,{\rm nm}$ for the side-chain of the oligolysines, based on Ref.[S3]

All charged particles (small ions, ionized side chains of PMAA and oligolysines) interacted via the Coulomb potential

$$U_{\rm Coulomb}(r) = k_{\rm B}T z_i z_j \frac{\lambda_{\rm B}}{r_{ij}} \tag{4}$$

where z is the charge number and r is the distance between the charges i and j. The solvent was implicit and it was included in the definition of the Bjerrum length

$$\lambda_{\rm B} = e^2/(4\pi\epsilon_0\epsilon_r k_{\rm B}T). \tag{5}$$

where ϵ_0 is the vacuum permittivity and ϵ_r is the relative permittivity of the solvent, which in the case of water at 298 K is $\epsilon_r = 78.5$. We set $\lambda_{\rm B} = 0.71\,{\rm nm}$ which corresponds to aqueous solution at ambient temperature $T = 300\,{\rm K}$.

The electrostatic interactions were computed using the particle-particle particle-mesh

(P3M) method.[S4] The algorithm was tuned to the relative accuracy of 10^{-3}, using the automated tuning routine implemented in the software ESPResSo v4.1.4.[S5–S7]

1.2 Simulation method and protocol

We simulated the systems using a combination of Langevin dynamics to sample the configurational space and Monte Carlo (MC) titration moves in the constant-pH ensemble[S8] to sample the acid-base equilibrium of the amine groups on the oligolysines. One simulation cycle consisted of 25 reaction trial moves, followed by 5000 integration steps of the Langevin dynamics. The Langevin dynamics simulations were performed using a time step $\delta t = 0.01\tau$, and a damping constant $\gamma = 1.0/\tau$, where $\tau = \sigma\sqrt{m/\epsilon}$. The particle mass m is arbitrary and has no effect on thermodynamic properties at equilibrium.

In each reaction trial move, the chemical identity of one side chain bead is changed following (1) switching its charge accordingly. These titration moves were coupled with the creation or deletion of a cation coion in order to maintain the electroneutrality of the system. The probability of accepting a titration trial MC titration move reads

$$P_{\mathrm{cpH}} = \min\left[1, \exp\left(-\frac{\Delta U}{\mathrm{k_B}T} + \xi\ln(10)\,(\mathrm{pH} - \mathrm{p}K_{\mathrm{a}})\right)\right], \tag{6}$$

where ΔU is the change in the potential energy of the system due to the trial step, usually dominated by electrostatic interactions, and ξ is the extent of the protonation such as $\xi = +1$ if a group is being protonated and $\xi = -1$ otherwise. In Eq. 6 we have not corrected for the excess chemical potential of the coion inserted or deleted in the simulation box.[S9] For dilute solutions of polyelectrolytes this omission has a small impact on the measured quantities which should be insignificant for the presented results.[S10]

A typical simulation consisted of 6000 cycles, *i.e.*, $t_{\mathrm{sim}} = 3 \times 10^5\,\tau$ of Langevin dynamics time evolution and 1.5×10^5 reaction trial moves. The configurations for post-processing and computing observables were stored with time intervals $t_{\mathrm{coord}} = 50\,\tau$, and the first 30% of each

simulation run was discarded as equilibration. The 30% threshold was chosen arbitrarily, so that it significantly exceeded the typical relaxation times and the equilibration times estimated by visual inspection, while simultaneously omitting this data did not significantly affect the estimated statistical uncertainty. Statistical uncertainty of the computed averages were estimated using the block analysis method, as described in Ref.[S11] The quality of sampling was assessed based on two slowly evolving observables: (1) the end-to-end distance and (2) the attachment and detachment of lysines at the polymer chains. The total simulation time was typically at least 70 times longer than the autocorrelation time of the end-to-end distance of the polymer, which was also estimated using the block analysis. This threshold was not reached in some simulations of Lys_8 at pH ≈ 11, which is reflected by slightly greater statistical uncertainty of the computed ensemble averages. Thus, the chains were given enough time to relax, so that the conformational statistics could be sufficiently sampled. The attachment and detachment events were assessed by visually inspecting the time evolution of the distance between individual lysine chains and the PMAA chain, as shown in Fig. S3. This figure shows that individual lysine molecules repeatedly switch between the states when they are attached to the polymer chain (distances around 1 nm), or detached (distances around 10 nm). Even in situations when the lysines are predominantly attached, they occasionally switch to the other state. This is particularly important in the case of Lys_8, which exhibited rather slow attachment/detachment dynamics.

Finally, to ensure that the attached lysines remained mobile, we monitored their position along the chain. Their mobility is confirmed by Fig. S4 which shows that their positions along the chain are rather homogeneously distributed. A detailed inspection of Fig. S4 reveals that, under the conditions when most lysines are detached, they are more often found closer to chain ends. This is what one would expect if the position of lysines is completely uncorrelated with the chain. On the contrary, when the lysines were mostly attached, they were more likely to be found closer to the central part of the chain. This can be understood by recognizing that the electrostatic attraction in the central part of the chain is stronger

than at the ends. Ultimately, Fig. S3 and Fig. S4 confirm that the configuration space of the studied system is reasonably well sampled and the system also makes occasional excursions to those parts of configuration space which have a low statistical weight.

1.3 The distribution of lysines and H^+ ions around PMAA

To complement the analysis of the different populations of lysines observed in our constant pH (cpH) simulations presented in the main text (Fig. 3), we have measured the distribution of lysines and H^+ ions around the PMMA chain.

In the case of lysine, we have directly sampled the average number of lysine chains as a function to the nearest bead of PMAA using a histogram procedure. This average number has been normalized by the total number of lysine chains in solution to calculate the mole fraction of lysine as a function of the nearest bead of PMAA, which is presented in Fig. S1.

The local concentration of H^+ ions, sometimes termed the "local pH", could be used as an alternative way of explaining the effect of PMAA on the ionization of lysines. In Fig. S2, we show how the local value of $c_{\mathrm{H}^+}(r)$ varies as a function of distance from PMAA for different pH values and length of the lysine chains, as determined from our simulations. We could not directly sample the distribution of H^+ ions because they are represented only implicitly in constant-pH simulation method.[S8] However, within the level of description of our computational model, H^+ ions have the same properties as any other small cation in solution. For this reason, the radial distribution function of the H^+ ions from any of the beads of PMAA must be the same as that of the Na^+ ions, *i.e.* $g_{\mathrm{H}^+,\mathrm{PMAA}}(r) = g_{\mathrm{Na}^+,\mathrm{PMAA}}(r)$. Since salt ions are explicit in our cpH simulations, we directly sampled $g_{\mathrm{Na}^+,\mathrm{PMAA}}(r)$ which we used to estimate the the local concentration of H^+ $c_{\mathrm{H}^+}(r)$ at a given distance to the PMAA chain r as

$$c_{\mathrm{H}^+}(r) = c_{\mathrm{H}^+}^{\mathrm{bulk}} g_{\mathrm{Na}^+,\mathrm{PMAA}}(r) = g_{\mathrm{Na}^+,\mathrm{PMAA}}(r) \times 10^{-\mathrm{pH}} \tag{7}$$

where we have approximated the concentration of H^+ in the bulk as $c_{\mathrm{H}^+}^{\mathrm{bulk}} = 10^{-\mathrm{pH}}$, corresponding to the ideal dilution limit in which the activity of proton is equal to its concentration, *i.e.* $a_{\mathrm{H}^+} = c_{\mathrm{H}^+}^{\mathrm{bulk}}$.

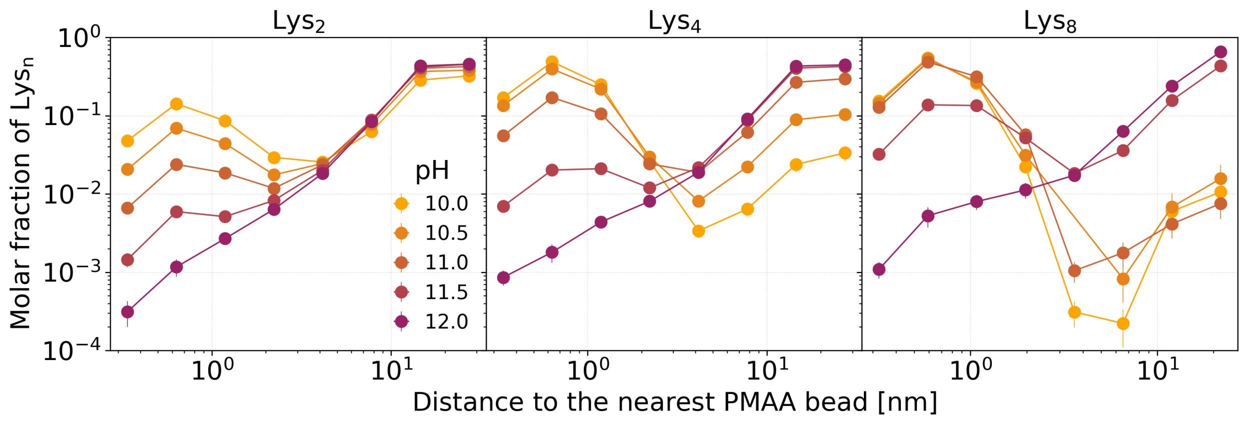

Figure S1: The mole fraction of Lys_n as a function of distance to the nearest PMAA monomeric unit, obtained from cpH simulations.

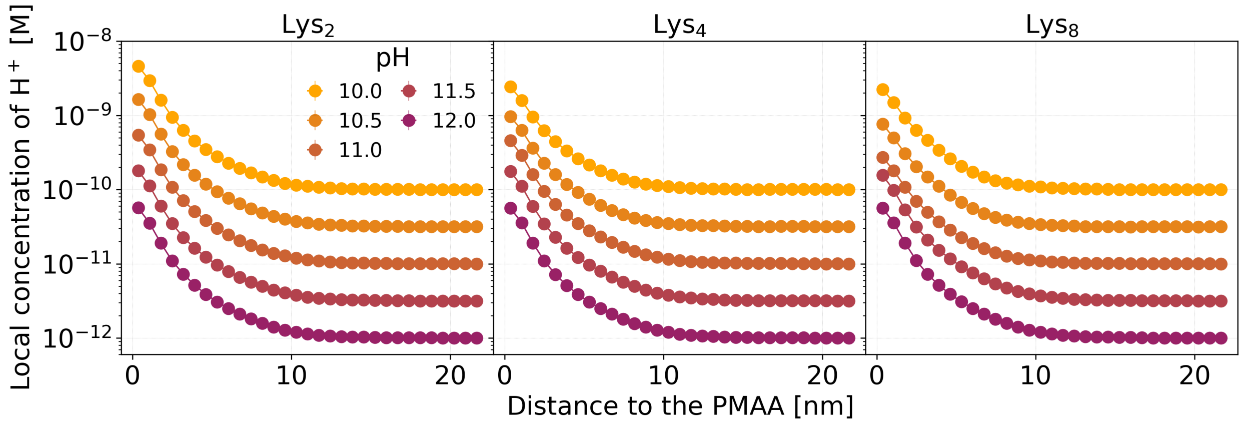

Figure S2: The local concentration of H^+ ions as a function of distance from the PMAA chain, computed from the cpH simulations.

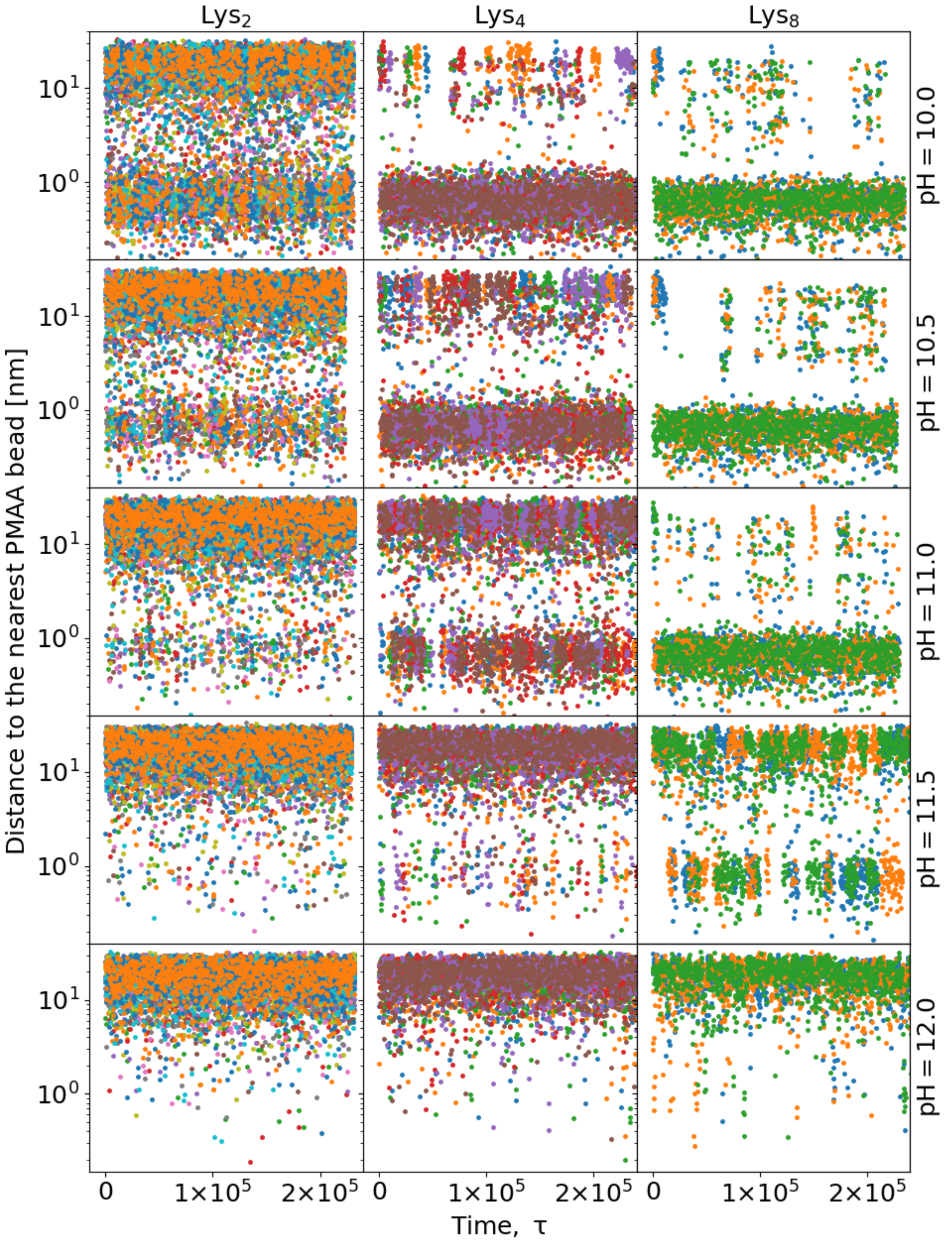

Figure S3: Time evolution of the distance between the Lys$_n$ and the nearest bead of the PMAA, obtained from simulations. Different colors represents the different oligolysine molecules within each system. These plots demonstrate that during each simulation the lysines were found attached to the polyeletctrolyte chain, as well as detached from the chain, which is the necessary condition for sufficient sampling of the configuration space.

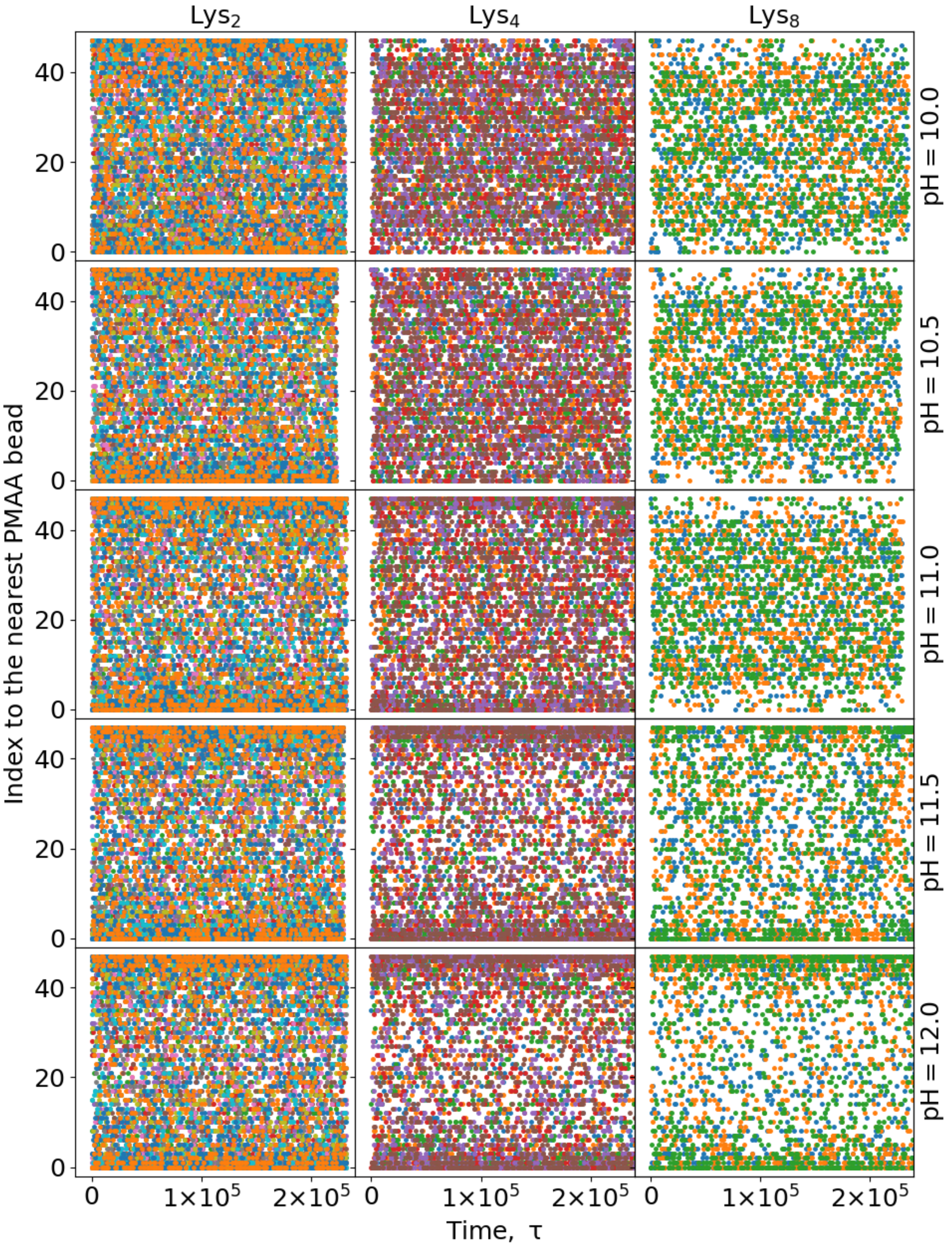

Figure S4: Time evolution of index of the nearest monomer to the peptide, showing that the condensed peptides remain mobile along the polymer backbone. Different colors represents the different oligolysine molecules within each system. These plots demonstrate that the attached lysines are homogeneously distributed along the polyelectrlyte chain, *i.e.*, they are not fixed to a particular position at the chain.

1.4 Simulation snapshots

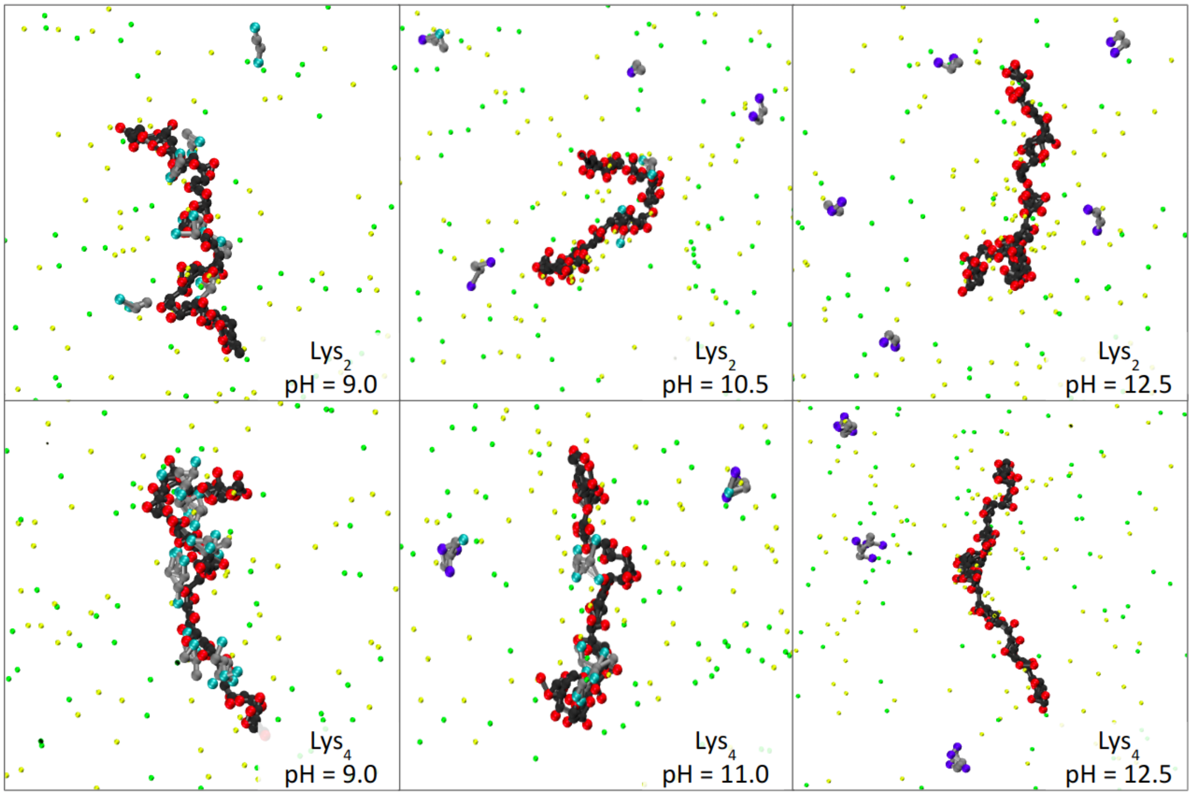

Figure S5: Simulation snapshots of the Lys_2 (top row) and Lys_4 (bottom row) interacting with PMAA. At pH = 9.0 ($\lesssim pK_A^{eff}$) the lysines are highly ionized and condensed on the PMAA chain, at pH = 12.5 ($\gtrsim pK_A^{eff}$) the lysines are weakly ionized and free in solution. However, at pH = 10.5 for Lys_2 and at pH = 11.0 for Lys_4 ($\approx pK_A^{eff}$) the two different ionization states coexist in the solution. Color code: gray = backbone groups; orange = non-ionized acidic groups; red = ionized acidic groups; blue = non-ionized basic groups cyan = ionized basic groups; green = small anion; yellow = small cation.

1.5 The effect of the PMAA chain length

To demonstrate that our conclusions are not significanlty affected by the chain length of PMAAA in simulations being much shorter than in the experiments, we performed additional simulations using $PMAA_m$ with $m = 96$, compared to the original simulations using with $m =$ 48. Simultaneously, we doubled the number of oligolysine molecules so that we maintained the same molar ratio of lysine to methacrylic monomeric units (1:2). In Fig. S6a we show that the ionization response of oligolysines as a function of pH is practically identical for

both PMAA chain lengths and all lengths of Lys_n. Similarly, Fig. S6b shows that the trend in the swelling of the PMAA is very similar in both sets of simulations. To facilitate the comparison of end-to-end distances of PMAA chains with different number of monomeric units, we scaled each end-to-end distance by the maximum value, observed in our simulations. Notably, although we doubled the computational time when simulating longer PMAA chains, the statistical quality of these results is worse than that of the simulations of shorter chains. Finally, we can conclude that the chain length used in this work ($m = 48$) was sufficient to obtain correct results and simultaneously convenient in terms of computational demands.

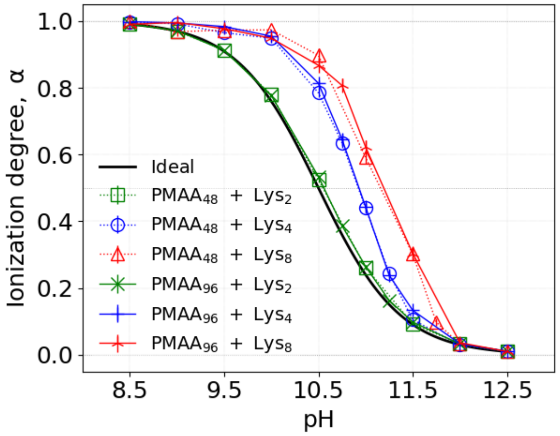

(a) Degree of ionization of Lys_n in presence of $PMAA_m$ with $m = 48$ (dashed lines) and $m = 96$ (solid lines).

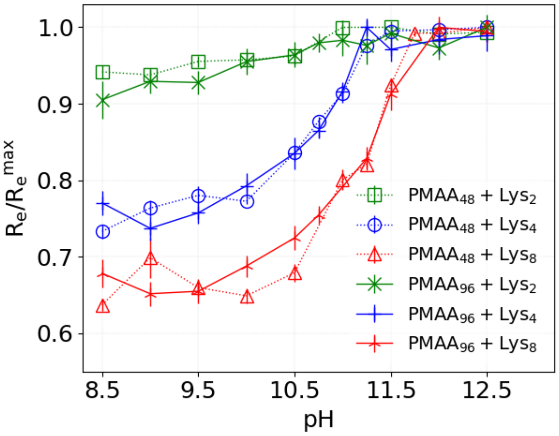

(b) Ratio between the end-to-end distance and the maximum observed end-to-end distance of $PMAA_m$ with $m = 48$ (dashed lines) and $m = 96$ (solid lines) in presence of Lys_n.

Figure S6: Simulation results comparing the systems with PMAA chains of different lengths, $m \in \{48, 96\}$, interacting with Lys_n with $n \in \{2, 4, 8\}$.

2 Experimental

2.1 Materials

Poly(methacrylic acid), PMAA, (100 $kg mol^{-1}$, Lot Number: 707334) was purchased from Polysciences Europe GmbH. Oligolysines with acetyl and amide terminal group and trifluoroacetate (TFA) as counterion were purchased from Biomatik Corporation, Kitchener, Ontario: Lys_2, Ac−KK−NH_2 (315.41 $g mol^{-1}$, 80.11% HPLC purity, Lot Number: P220722 - XF148268), Lys_4, Ac−KKKK−NH_2 (571.76 $g mol^{-1}$, 97.75% HPLC purity, Lot Number: P220628 - LC349184), Lys_8, Ac−KKKKKKKK−NH_2 (1084.44 $g mol^{-1}$, 96.23% HPLC purity, Lot Number: P220628 - YW822069). All solutions in the experiments were prepared with HPLC grade water purchased from Sigma-Aldrich. Standard solutions of HCl, 100 mM, and NaOH 100 mM, from Carl Roth GmbH. Deuterium oxide, 99.8% purity and sodium trimethylsilylpropanesulfonate (DSS), 97.0% purity, from Sigma-Aldrich was used for field-frequency lock.

2.2 Potentiometric titration

We performed the potentiometric titrations on a Metrohm 888 Titrando Compact titrator equipped with a Pt1000 temperature sensor and a magnetic stirrer. The pH was measured using a LL Biotrode 3.0 mm glass electrode and the results were collected by Titrando Software. Sample preparation consisted in weighing 20.0 mg of each oligolysine and dissolving them independently in 4.0 mL of standardized HCl (0.1 M).

The initial titrate volume was 2.0 mL and the titrations were performed in duplicate until reaching a pH of 12.5 using standardized NaOH (0.1 M). The blank titration curve corresponds to titrate 2.0 mL of standardized HCl (0.1 M). All stock solutions were kept under soda lime to prevent CO_2 absorption.

2.2.1 Ionization response from potentiometric titrations

The raw data collected by the potentiometric titrations correspond to a set of temperatures, pHs and the titrant volume (NaOH) at different time intervals. Therefore, we processed this information to obtain the ionization degree α of each oligolysine as a function of the pH. First, we got the pK_w values corresponding to the registered temperatures. We also include the initial titrate volume (we assumed negligible the analyte volume, then $V_0 = V_0^{\mathrm{HCl}}$) into the set of titrant volumes in order to obtain the total volume (V_{total}) at each time interval. Using the pH and the pK_w values, we calculated the amount of H^+ and OH^- ions at each step of the titration as

$$n_z^{\mathrm{H}^+} = 10^{(-\mathrm{pH})} c^{\ominus} V_{\mathrm{total}}, \qquad n_z^{\mathrm{OH}^-} = 10^{(\mathrm{pH}-\mathrm{p}K_\mathrm{w})} c^{\ominus} V_{\mathrm{total}} \tag{8}$$

where the reference concentration is $c^{\ominus} = 1\,\mathrm{mol/kg}$ which can be approximated as $1\,\mathrm{mol/dm^3}$ in dilute aqueous solutions. We took the temperature-dependence of pK_w into account, following Ref.[S12] Additionally, we computed the number of charges contributed by the stock solution of HCl and the number of charges contributed by the titrant:

$$n_z^{\mathrm{HCl}} = V_0^{\mathrm{HCl}} \cdot c_{\mathrm{HCl}}, \qquad n_z^{\mathrm{NaOH}} = \cdot V^{\mathrm{NaOH}} \cdot c_{\mathrm{NaOH}} \tag{9}$$

Knowing all the charges contributed by each constituent and the amount of oligolysine in the titrate, we calculated the charge per lysine unit,

$$Z_{\mathrm{Lys}} = (-1) \cdot \frac{(n_z^{\mathrm{NaOH}} - n_z^{\mathrm{HCl}} + n_z^{\mathrm{OH}^-} - n_z^{\mathrm{H}^+})}{n_{\mathrm{Lys}}} + Z_{\mathrm{extra}} \tag{10}$$

where Z_{extra} is the amount of extra charge from the excess triflouroacetate counterions which come from the synthesis of oligolysines. The value of Z_{extra} was determined empirically by shifting the resultant curves so that the computed charge per lysine unit in the plateau at low pH corresponds to +1.

2.2.2 Titration of free lysines

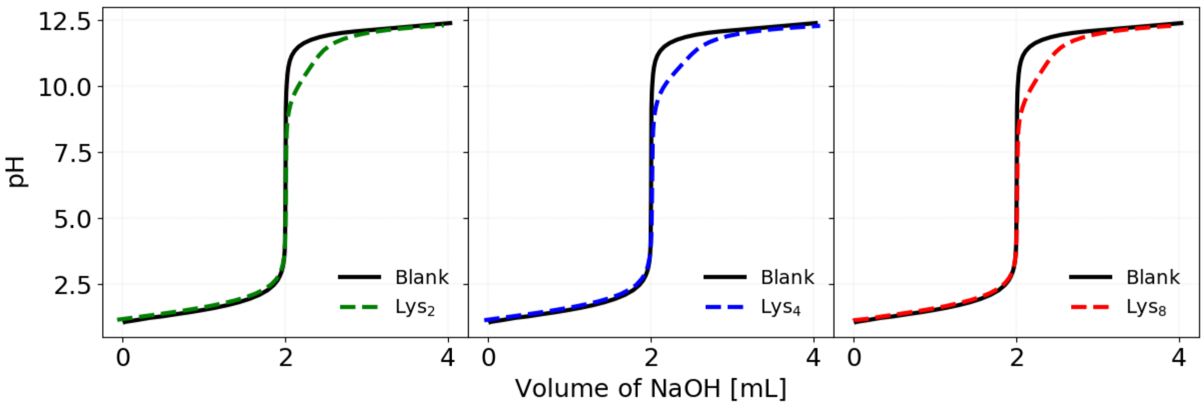

Figure S7: Titration curves of Lys_n in absence of PMAA.

Fig. S7 shows the titration curves of Lys_n, compared to the titration curve of the blank.

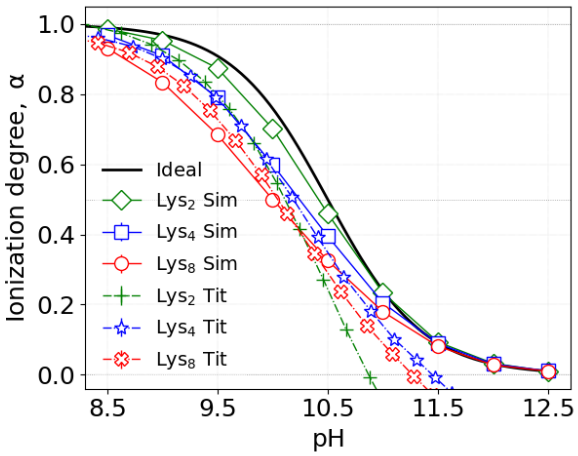

Figure S8: Degree of ionization of Lys_n in absence of PMAA as a function of pH. Simulations (squares and circles, respectively) and potentiometric titration results (stars and crosses, respectively).

Fig. S7 shows the titration curves of Lys_2, Lys_4 and Lys_8, which are the primary outputs of potentiometric titration measurements. For reference, we also show the blank titration curve in each of the panels in Fig. S7. These curves have been processed using Eq. 10 to obtain the charge per lysine unit.

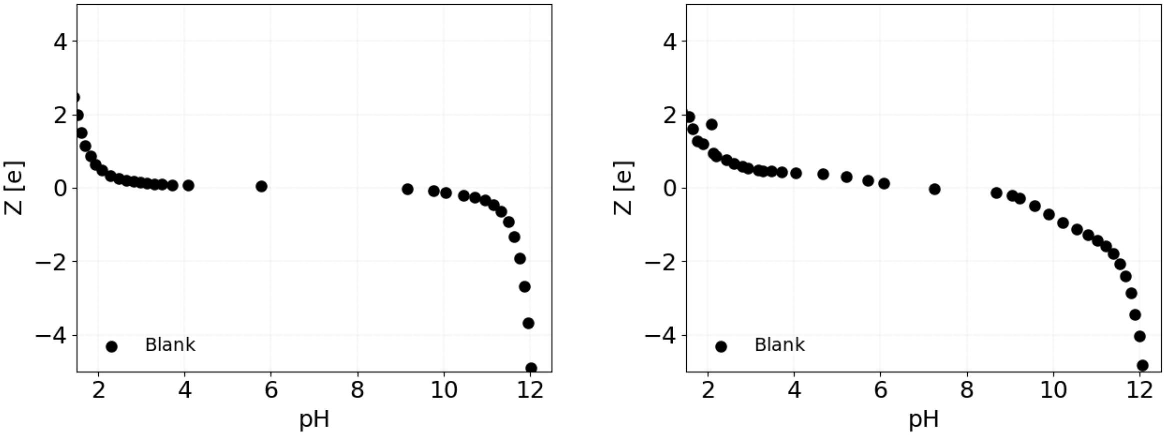

(a) In absence of CO_2. (b) In presence of CO_2.

Figure S9: Charge computed from potentiometric titration blank.

In Fig. S8 we compared the charge per lysine unit, computed from potentiometric titrations, to our simulation results. The comparison is nearly quantitative up to pH $\gtrsim$ 10.5, which confirms the suitablility of our model. At pH $\gtrsim$ 10.5, the absorption of CO_2 from the air affected the measurement, causing that the charge calculated from the titration was lower than the actual charge on the lysines. At pH $\gtrsim$ 11, the charge from the dissolved CO_2 overwhelms the actual charge on the lysines, causing that the charge calculated from potentiometric titrations attained a negative value. The titrations of Lys_2 did not yield plausible results, presmably because its effective pK_A was too close to 10.5, so that the CO_2 interfered with the measurement. Therefore, we excluded Lys_2 from further discussion and for the other lysines we considered only the values at pH $\lesssim$ 10.5.

2.3 Nuclear magnetic resonance spectroscopy

2.3.1 Preparation of samples

The samples of PMAA + Lys_8 for NMR measurements were prepared as follows:

1. PMAA was dissolved in 10mM NaOH to obtain a solution of polymer concentration $c = 18\,\mathrm{g/l}$). Then, 1M HCl was carefully added to adjust the pH to pH ≈ 7

2. Lys_8, was dissolved in 10mM HCl to obtain a solution with concentration 18 g/l. Then, 1M NaOH was carefully added to adjust the pH to pH ≈ 7

3. The solution of Lys_8 was added to PMAA solution to obtain the molar ratio of ionizable groups [Lys]/[MAA]=0.5 and to achieve the final PMAA concentration $c = 15$ g/l.

2.3.2 NMR measurements

All NMR data were recorded using a Bruker AVANCE III spectrometer operating at the proton Larmor frequency of 600 MHz equipped with a cryogenically cooled probe and with temperature stabilized at 25 °C. Samples were prepared as H_2O solutions with a capillary insert containing D_2O with a trace of DSS that was used for field-frequency lock and chemical shift referencing. The ^1H spectra were acquired with water suppression using excitation sculpting method.[S13] Measurements of translational diffusion coefficients were performed with the double stimulated echo experiment with bipolar pulse field gradients described by Jerschow et al.,[S14] combined with water suppression. The gradients were 1.5 ms long with 32 linearly spaced amplitudes spanning the range 0 Gcm^{-1} to 60 Gcm^{-1}. The diffusion time was set either to 200 ms or 800 ms in two independent runs that focused either on fast or slow diffusing particles, respectively. The calibration was done using a standard sample of 1% H_2O in D_2O (doped with $GdCl_3$), for which the value of the HDO diffusion coefficient at 25 °C is $1.9 \times 10^{-9}\,m^2s^{-1}$. NOESY spectra were acquired using a mixing time of 100 ms, employing excitation sculpting to suppress the water signal.[S13] The ^{13}C NMR spectra were acquired using a standard method with continuous ^1H decoupling. The degree of ionization of lysine side chains was compupted from the chemical shifts of specific peaks in ^{13}C NMR spectra of Lys8 and Lys8-PMAA, which have been described in literature as good reporters of ionization.[S15]

The noise level in the spectra of samples containing PMAA indicates partial precipitation of the polymer. The precipitated polymer may not be visible in the NMR spectra, therefore, these results should be interpreted cautiously.

2.3.3 Ionization response from NMR

The degree of ionization was calculated from the chemical shift of the Carbon B in the lysine residue using Eq. 11.[S15]

$$\alpha(\text{pH}) = \frac{\delta_{max} - \delta(\text{pH})}{\delta_{max} - \delta(\text{min})} \tag{11}$$

2.3.4 13C NMR spectra of Lys_8 and PMAA + Lys_8 at various pH values

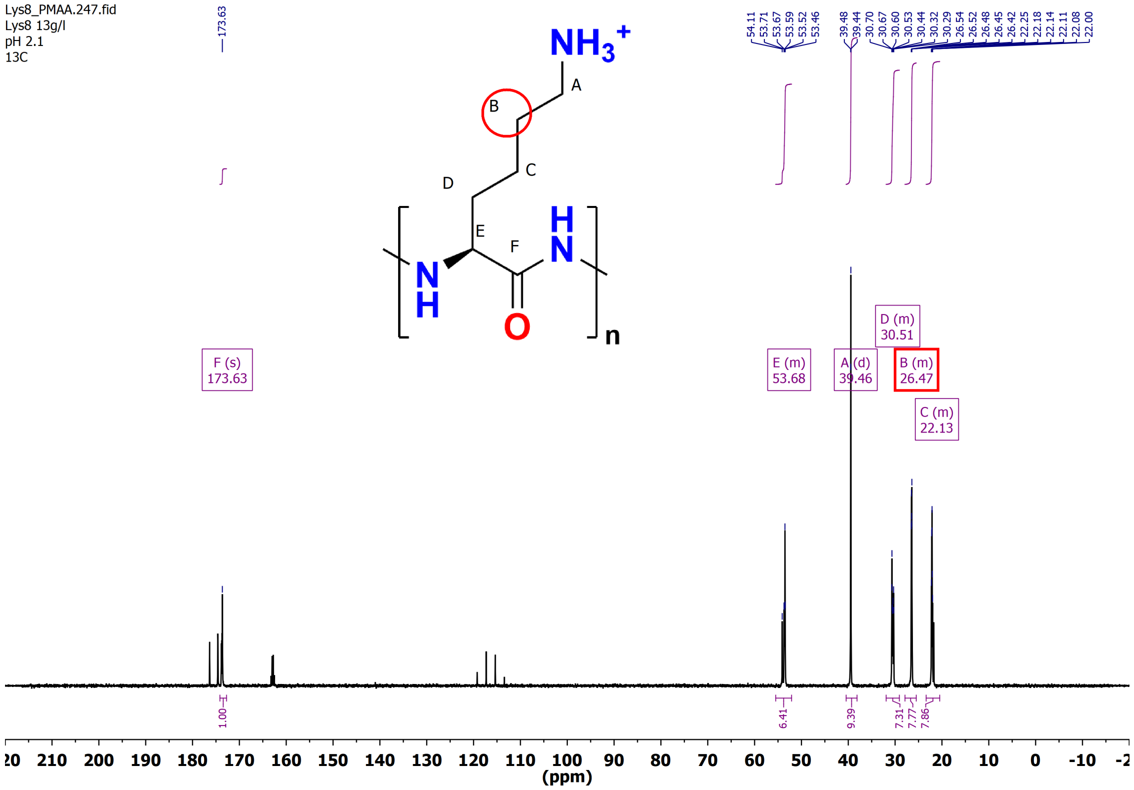

Figure S10: ^{13}C NMR spectra of Lys_8 at various pH values

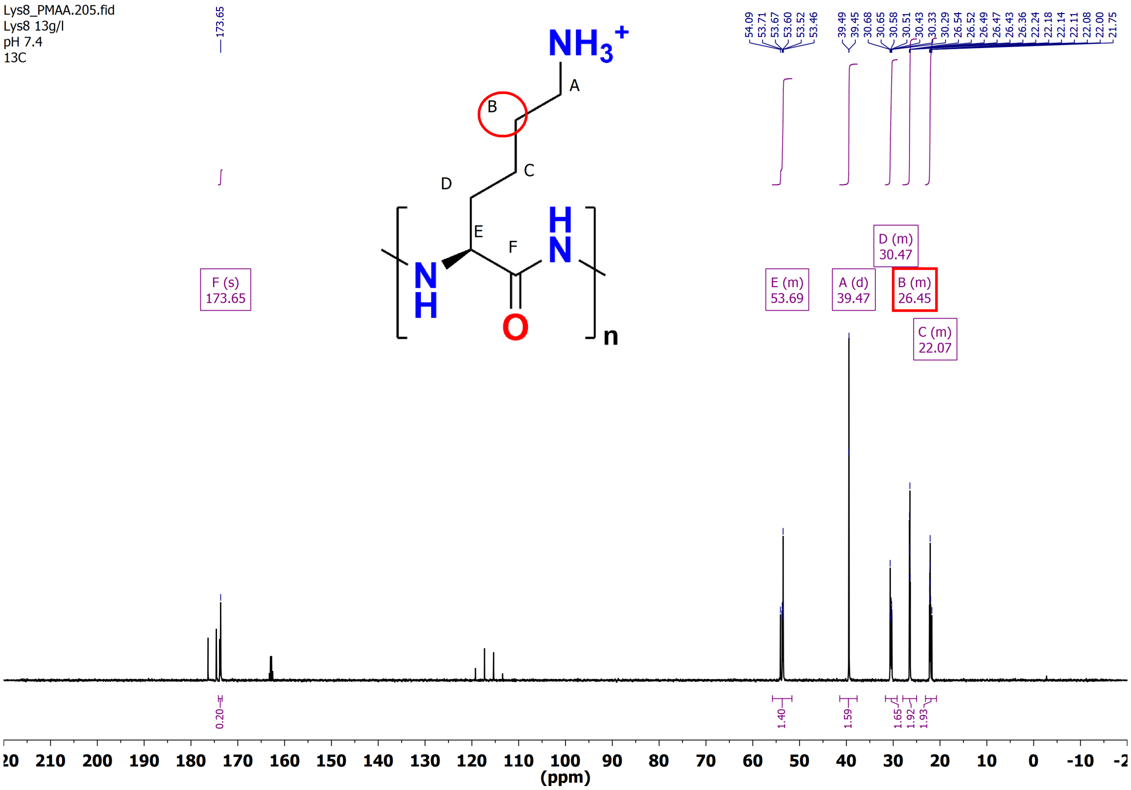

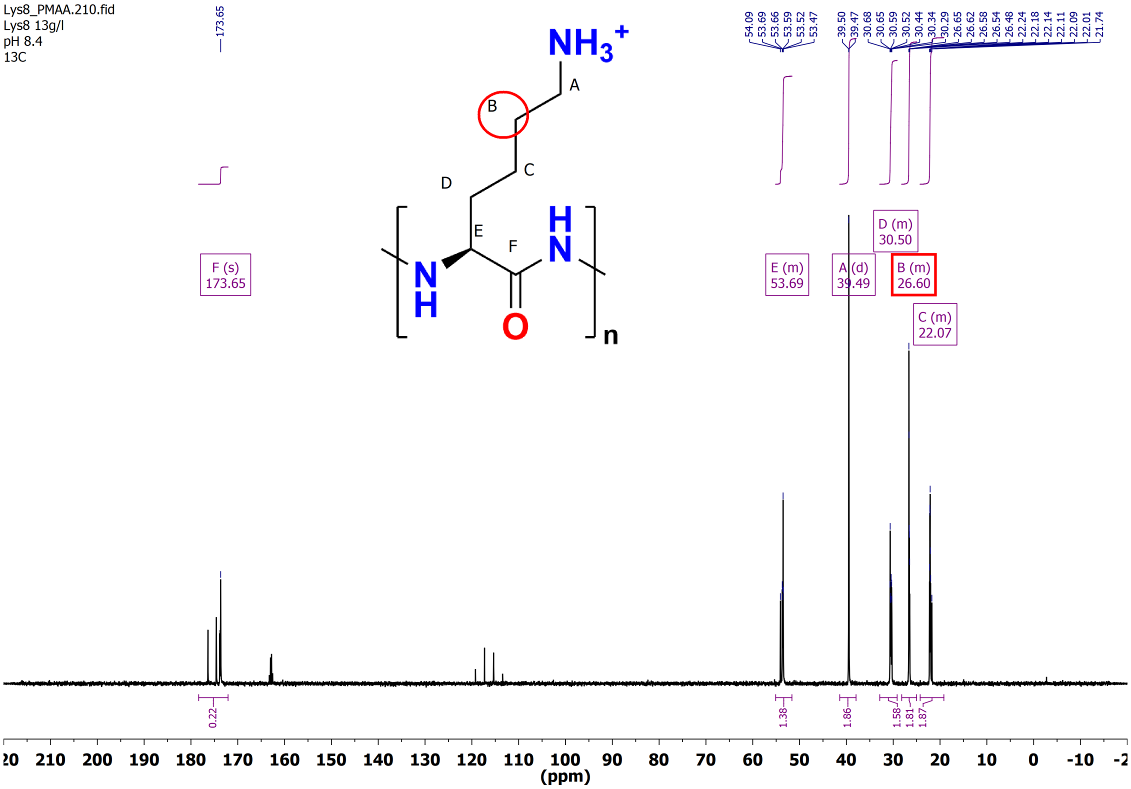

Figure S10: (continued) ^{13}C NMR spectra of Lys_8 at various pH values

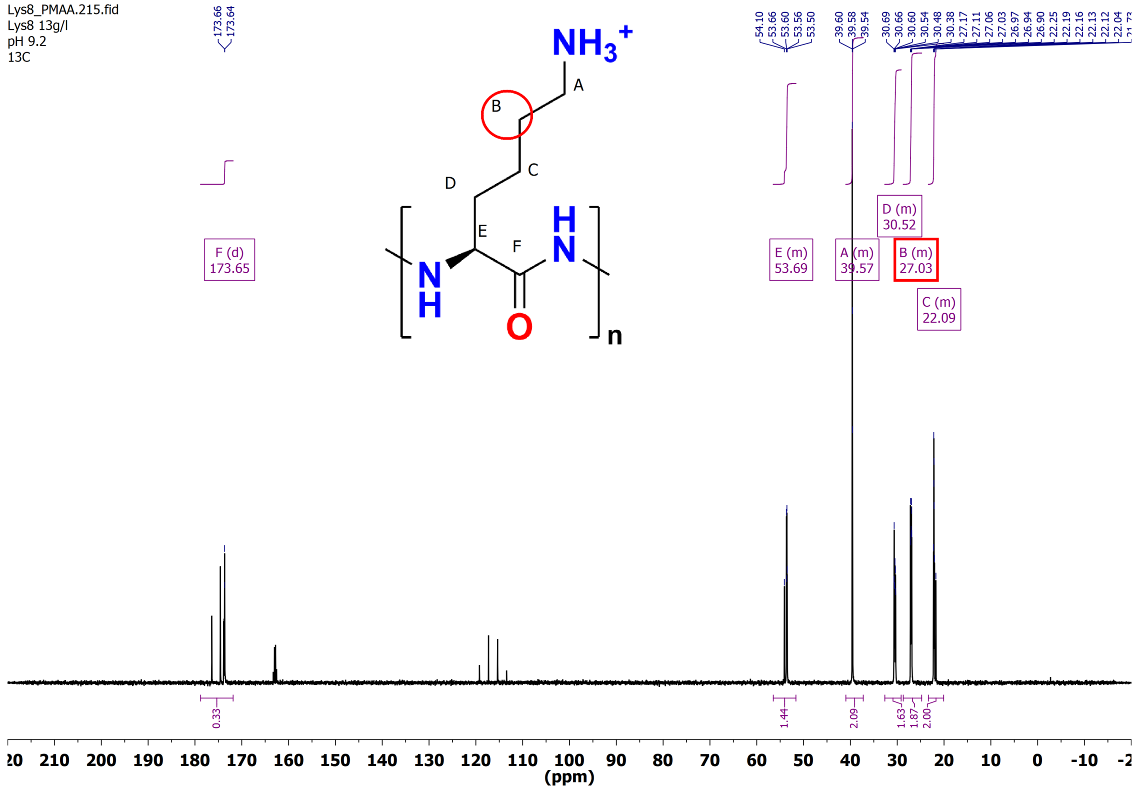

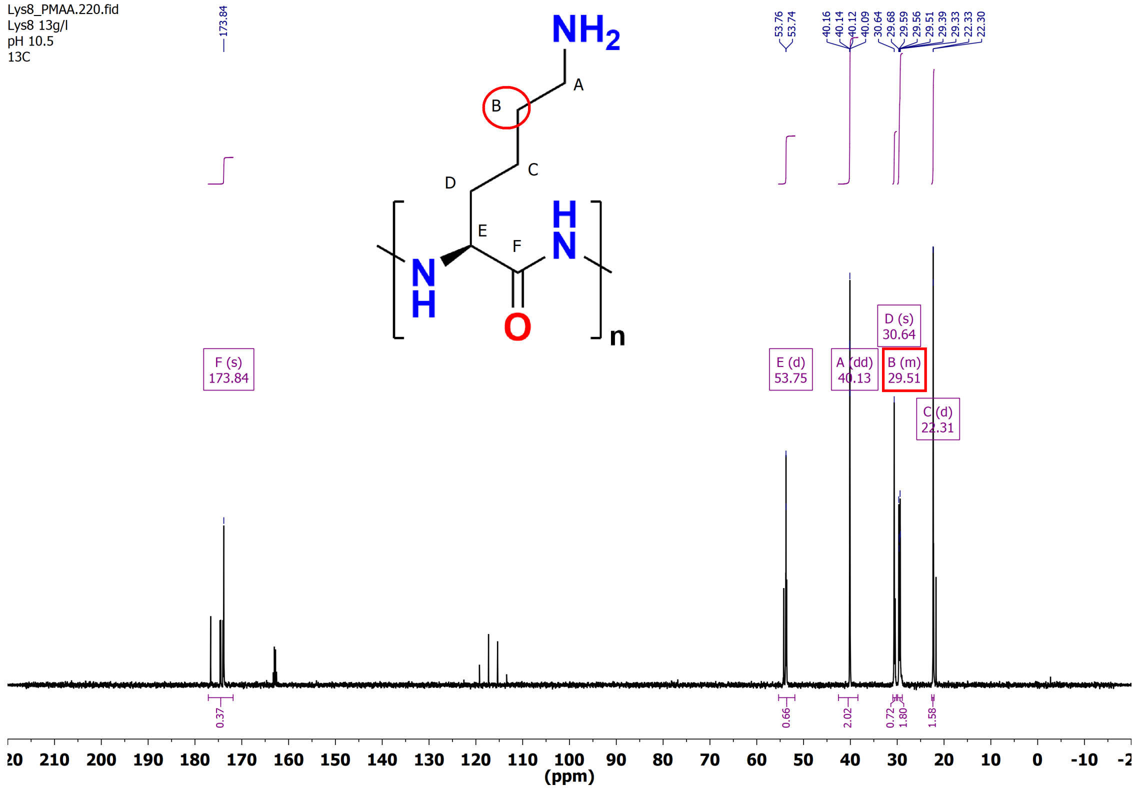

Figure S10: (continued) ^{13}C NMR spectra of Lys_8 at various pH values

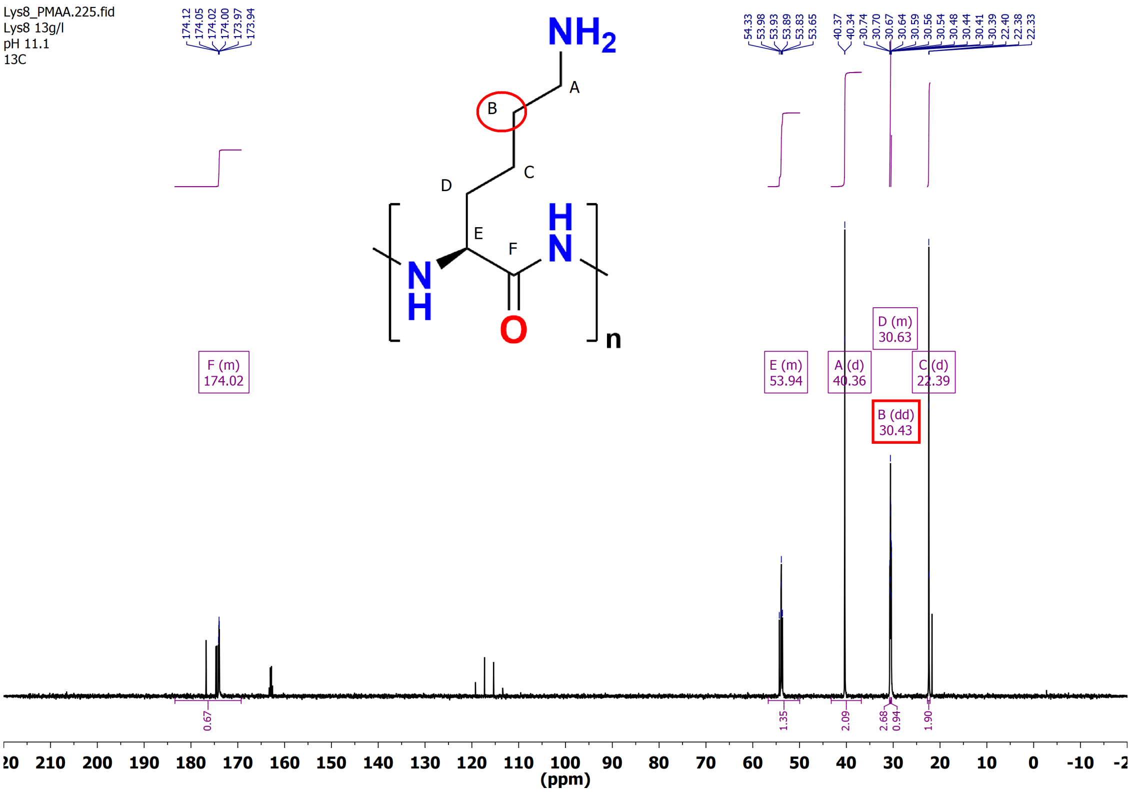

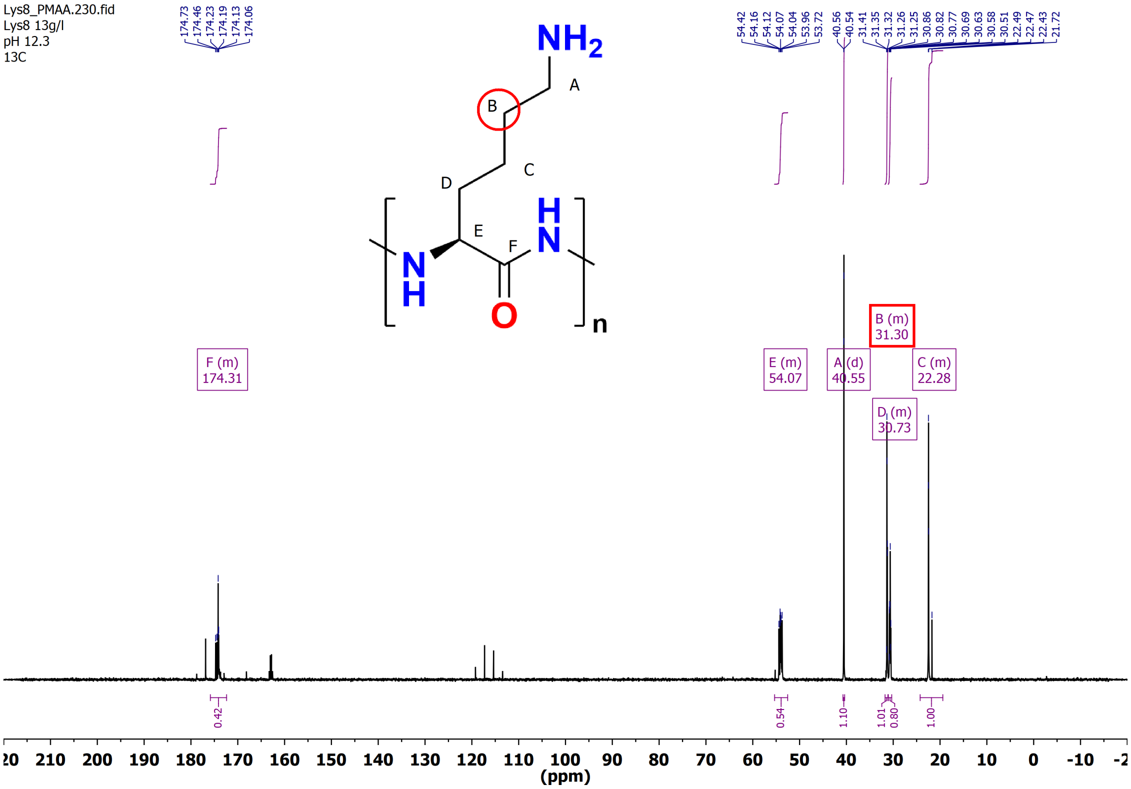

Figure S10: (continued) ^{13}C NMR spectra of Lys_8 at various pH values

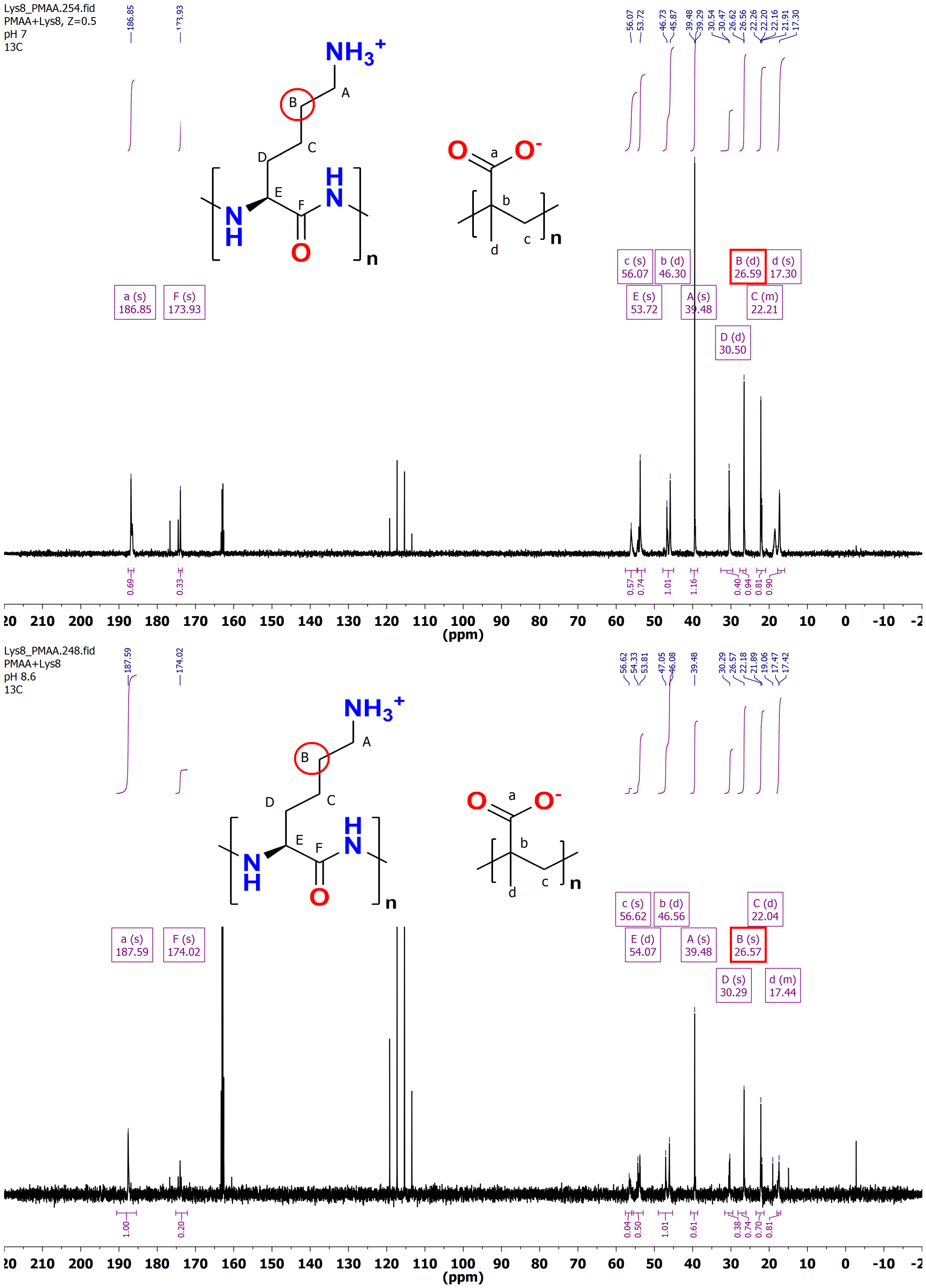

Figure S11: ^{13}C NMR spectra of PMAA + Lys_8 at various pH values

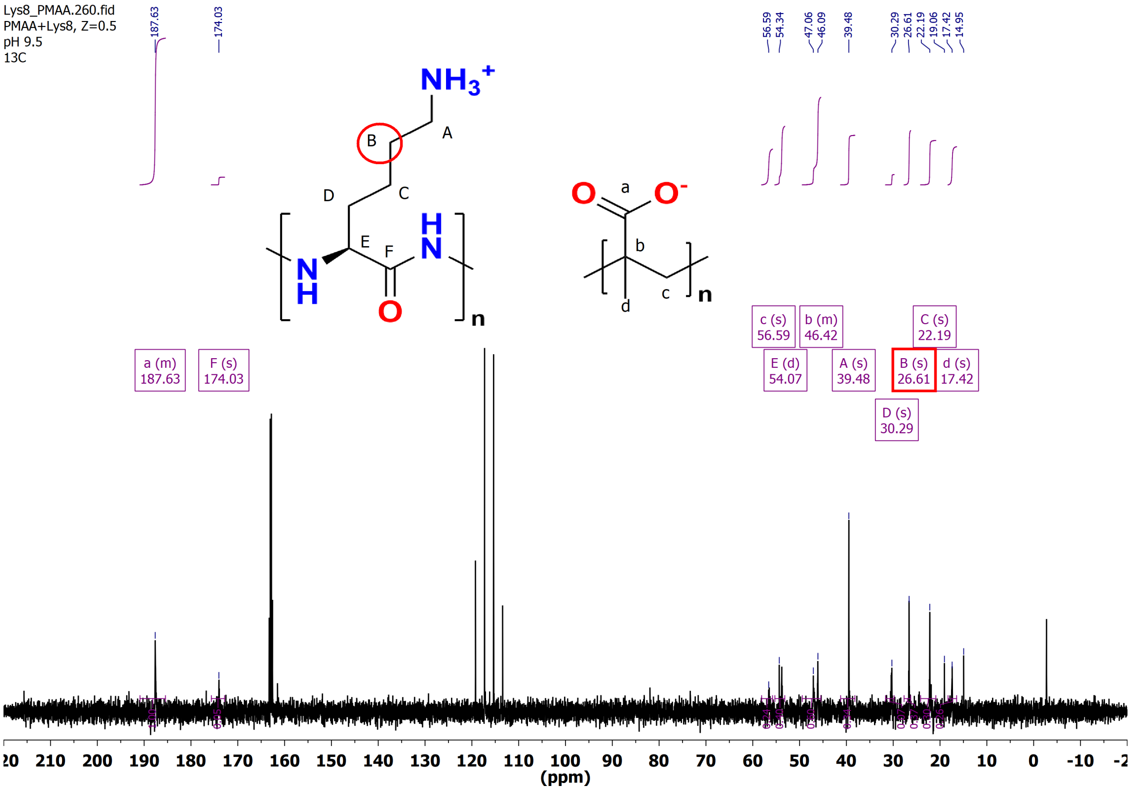

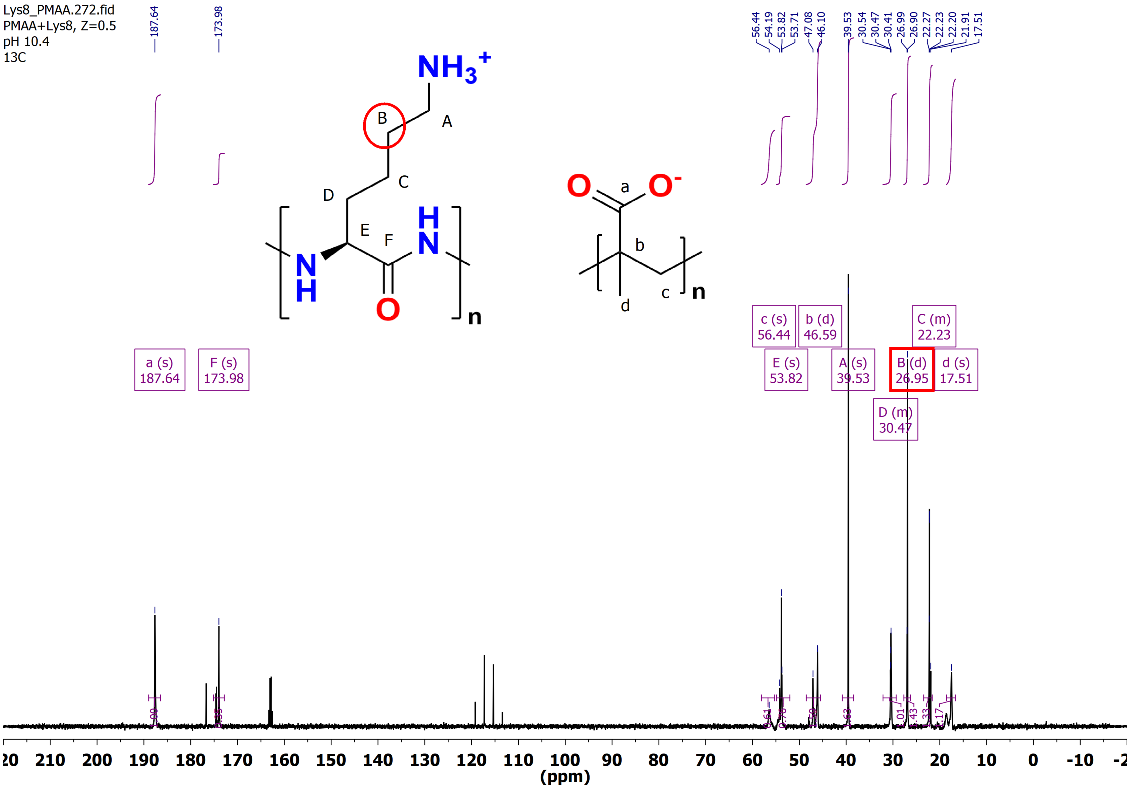

Figure S11: (continued) ^{13}C NMR spectra of PMAA + Lys_8 at various pH values

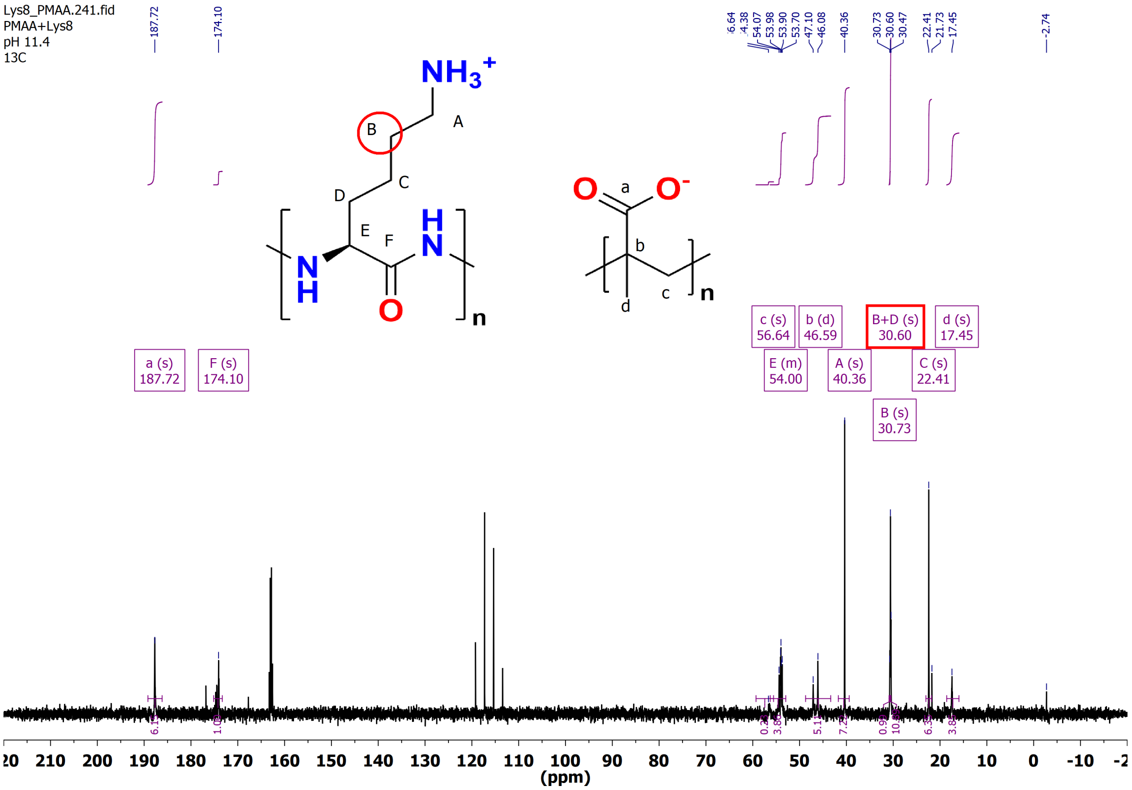

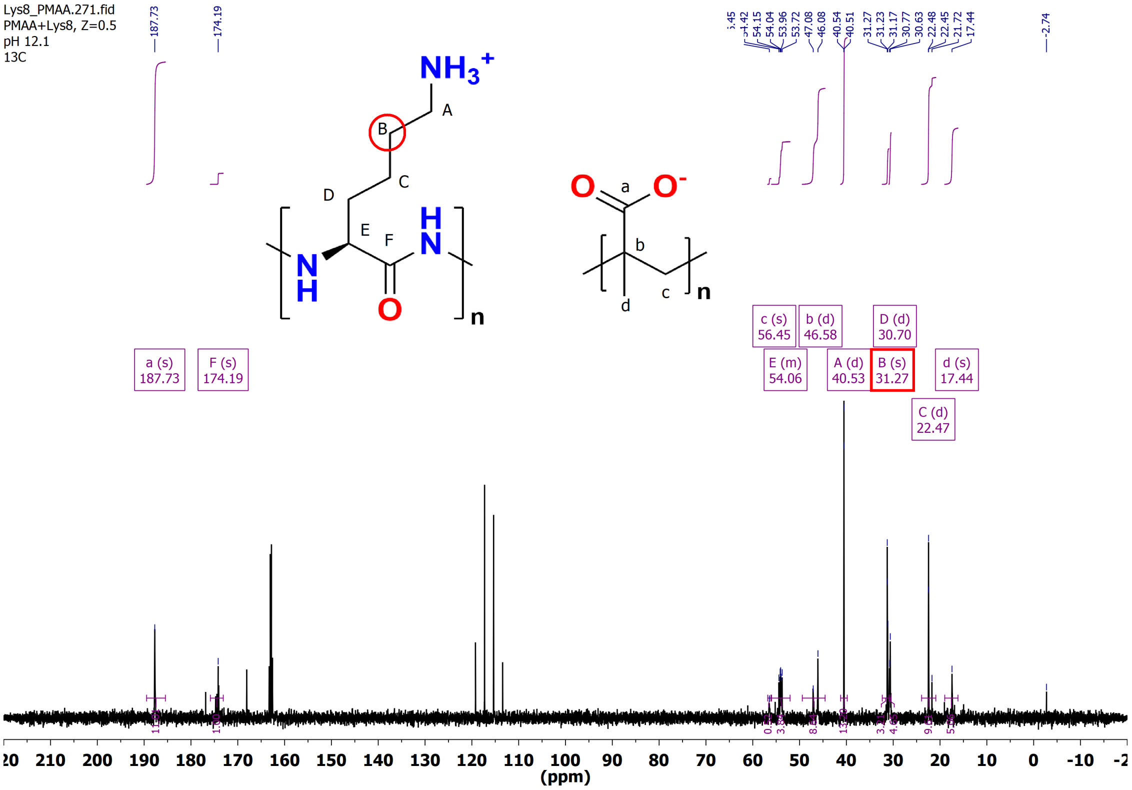

Figure S11: (continued) ^{13}C NMR spectra of PMAA + Lys_8 at various pH values

2.3.5 1 H NMR spectra of $Lys_8{}^+$ and Lys_8 + PMAA

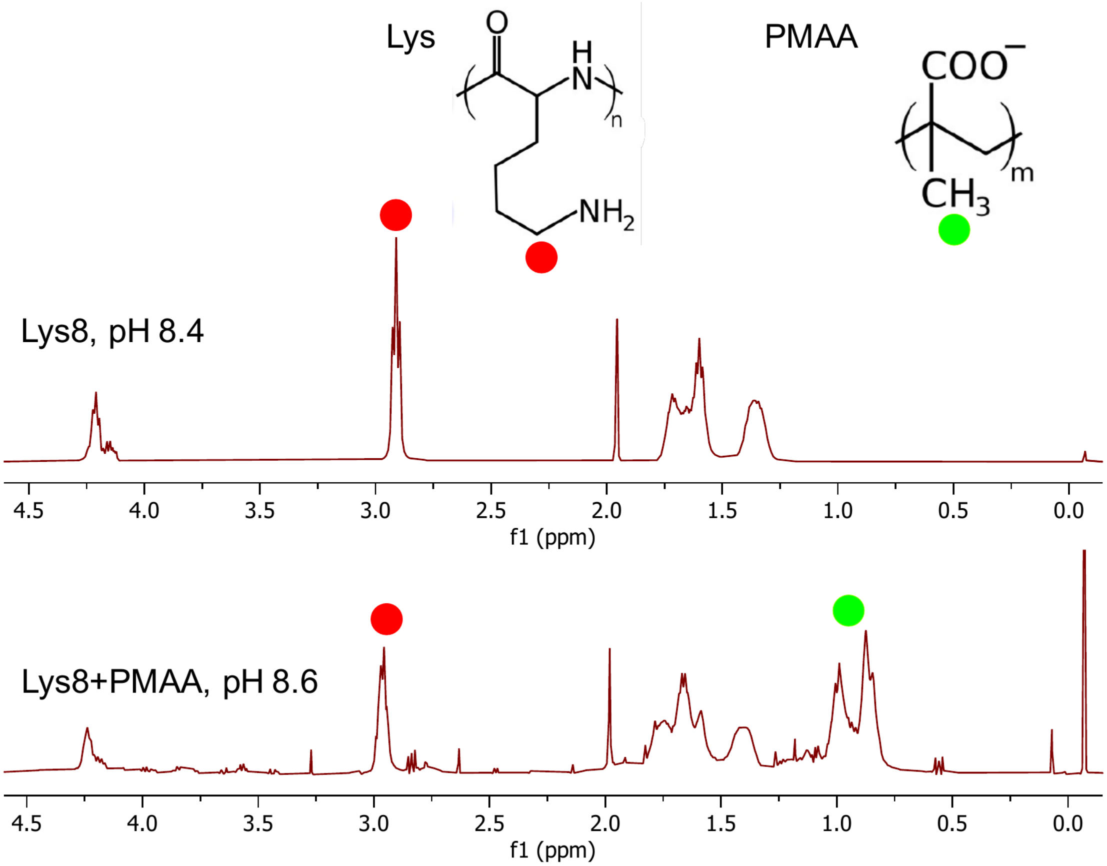

Figure S12: ^{1}H NMR spectra of Lys_8 and PMAA + Lys_8 at various pH values. The peaks of the CH_2 group on Lysine (red) and CH_3 PMAA (green) do not overlap and are thus used for the analysis in DOSY measurements.

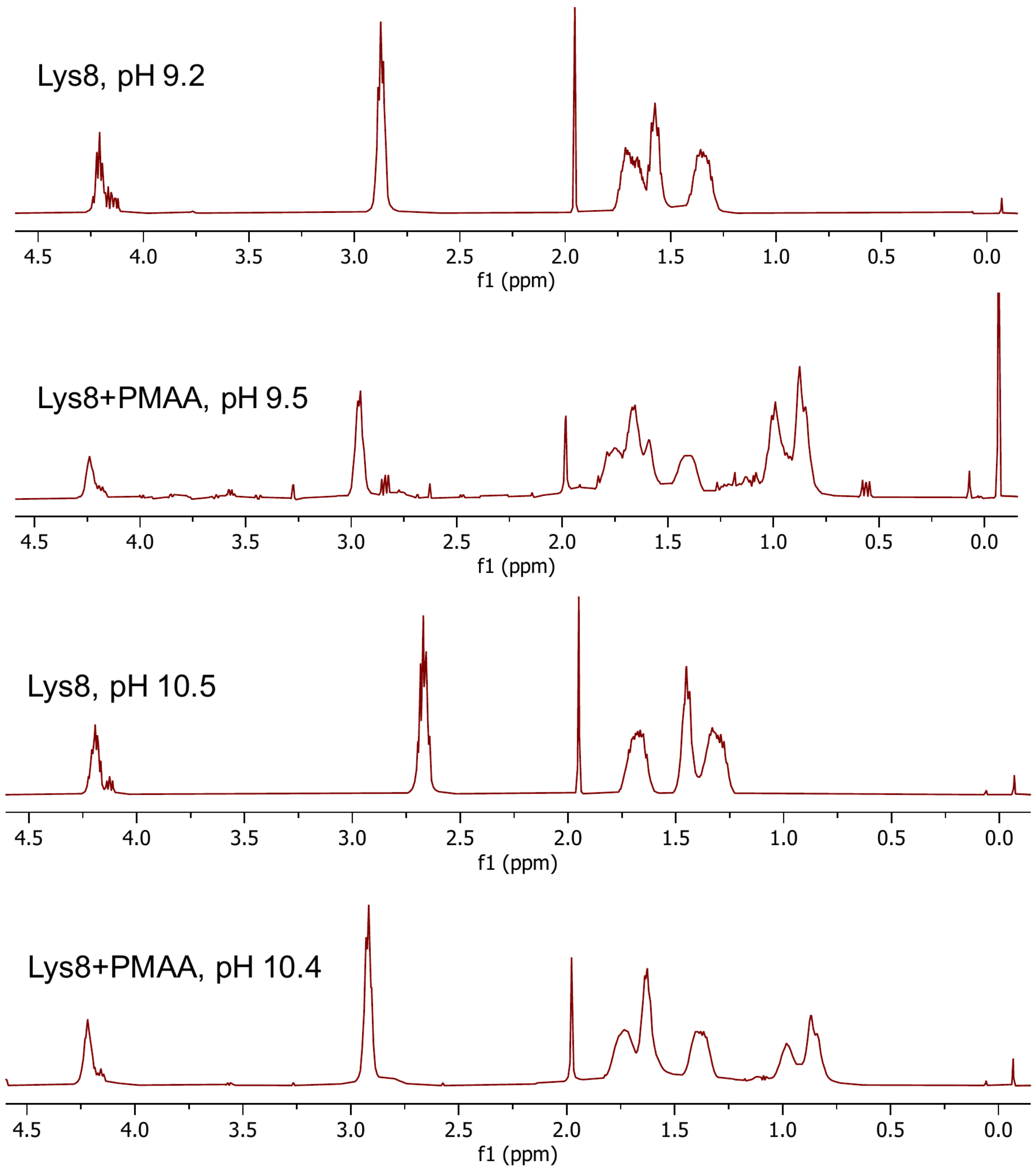

Figure S12: (continued) ^{1}H NMR spectra of Lys_8 and PMAA + Lys_8 at various pH values

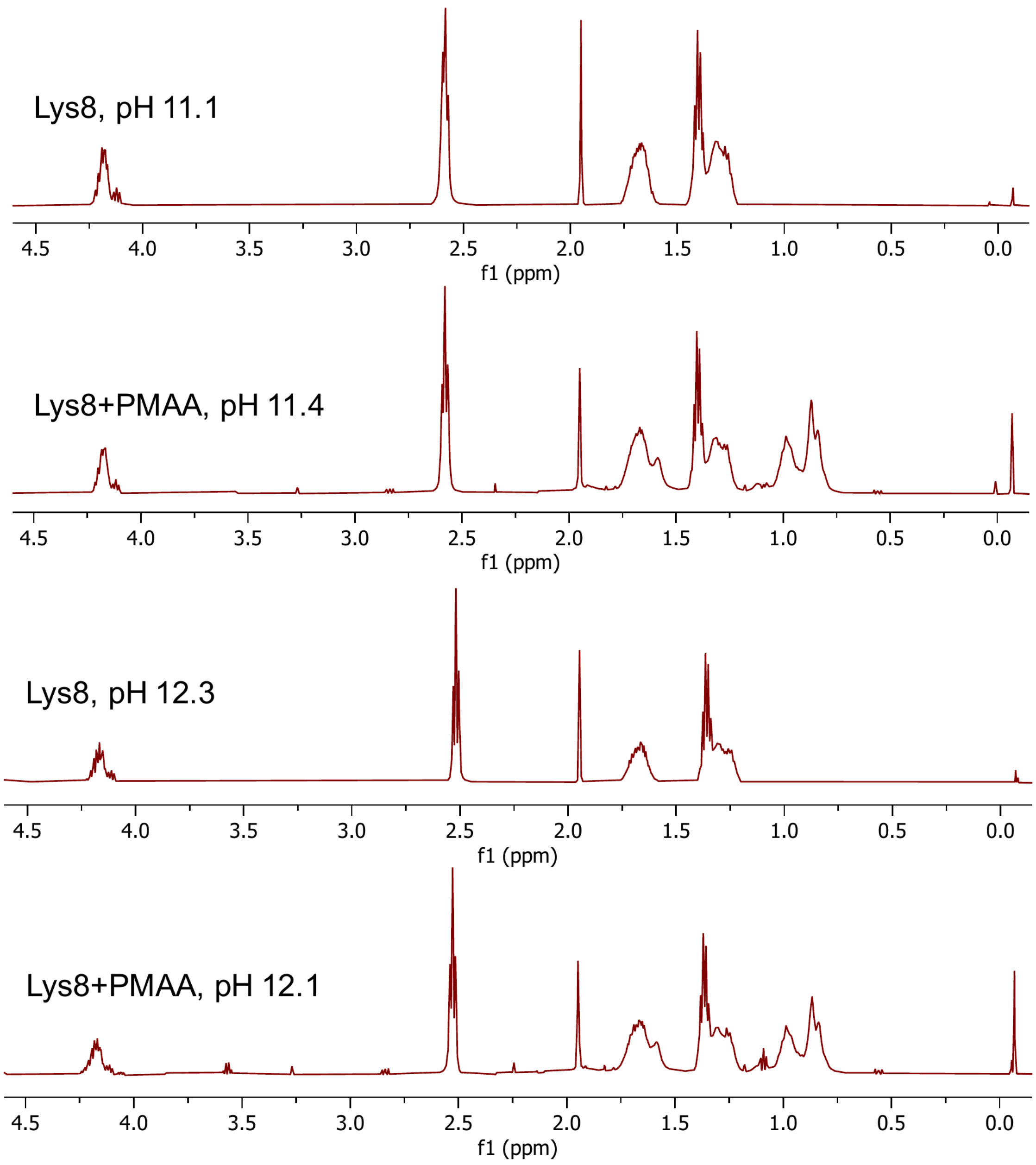

Figure S12: (continued) ^{1}H NMR spectra of Lys_8 and PMAA + Lys_8 at various pH values

2.3.6 Analysis of DOSY spectra of pure Lys_8

Integral intensity of Lys CH_2 peak at around 3ppm and of PMAA CH_3 peaks at around 1ppm were evaluated as a function of gradient strength. This dependency is described by the Stejskal-Tannor formula

$$I(g) = I_0 \exp\left\{-Dkg^2\delta^2\left(\Delta - \frac{\delta}{3}\right)\right\} \tag{12}$$

where D is the diffusion constant, δ is duration of the gradient pulses, Δ is time interval between encoding and decoding gradients (diffusion time). The constant k was calibrated using 5% H_2O in D_2O sample, assuming the diffusion constant of HDO to be $1.90 \times 10^{-9} m^2/s$. Diffusion constants of Lysine were obtained by fitting a single exponential to Lysine peak integrals. In case of the PMAA peak, a single exponential (grey dashed line in Fig. S14) or a sum of two exponentials (red lines in Fig. S14) were used, resulting in two diffusion constants and of the two components. At all pH values except 8.6 and 9.6, the double exponential fits describe the diffusion of PMAA significantly better than single exponential fits. For the PMAA, we can expect a range of diffusion coefficients, corresponding to chains of various lengths in the polydisperse sample. Detailed analysis of PMAA diffusion is not relevant in the current context. The diffusion coefficient of PMAA is needed only as a reference to confirm that the slow diffusion of oligolysines is on the same order of magnitude. Therefore, we used only the mean diffusion coefficient of PMAA, obtained from single-exponential fits.

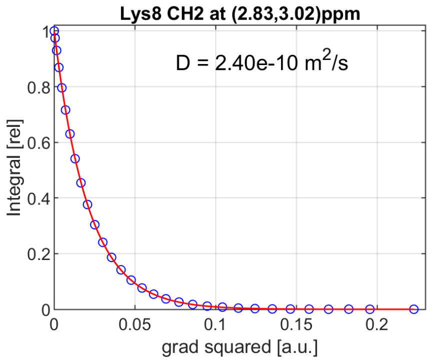

(a) pH = 7.4

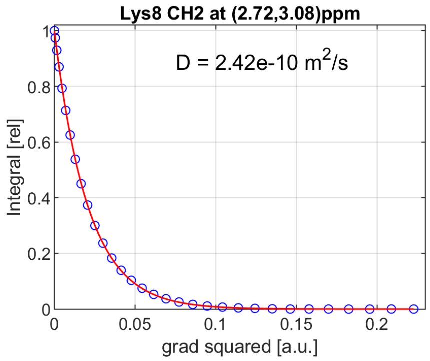

(b) pH = 8.4

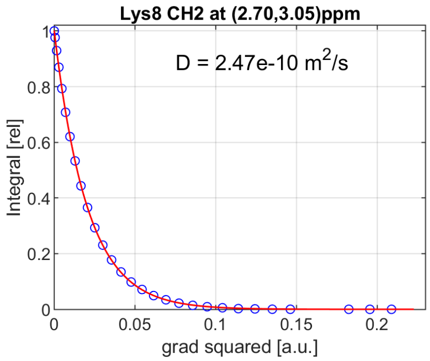

(c) pH = 9.2

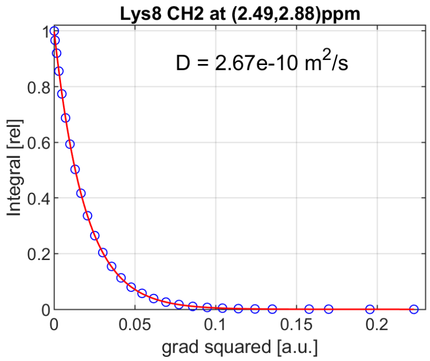

(d) pH = 10.5

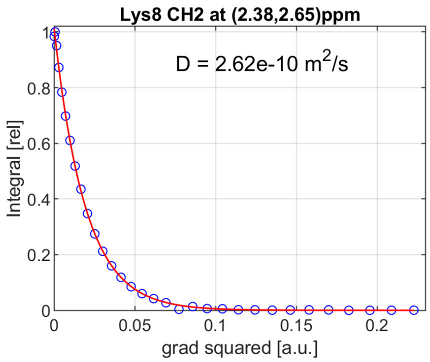

(e) pH = 12.3

Figure S13: Evaluation of DOSY spectra of pure Lys_8 at various pH values. The signal intensities were obtained as integral over the spectral region indicated in the title of each graph. Exponential fits were used to obtain the diffusion coefficient, the value of which is indicated in the inset.

2.3.7 Analysis of DOSY spectra of PMAA + Lys_8

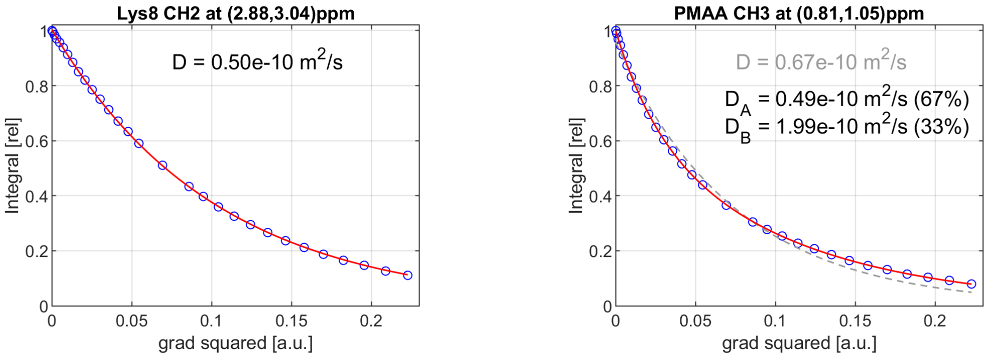

(a) pH = 7.0

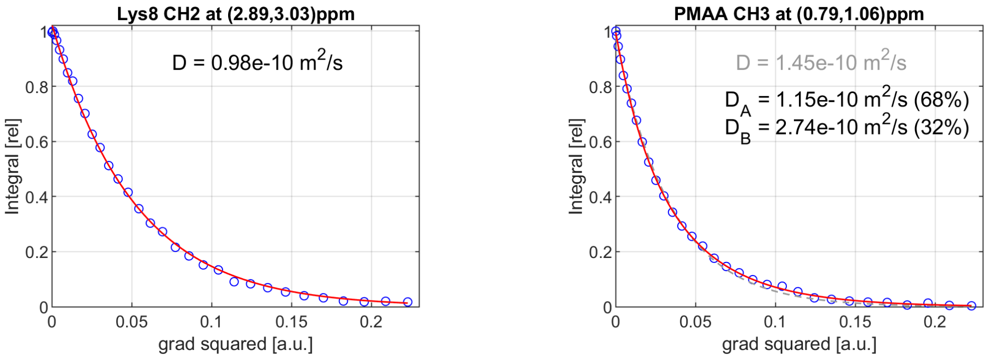

(b) pH = 8.6

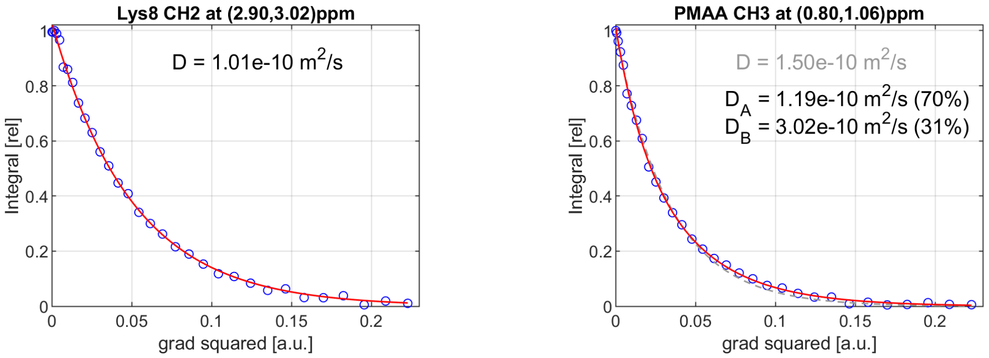

(c) pH = 9.5

Figure S14: Evaluation of DOSY spectra of Lys_8 (left) and PMAA (right) in the PMAA + Lys mixture at various pH values. The Lys8 dependencies were fitted using a single exponential. The PMAA dependencies were fitted using both a single exponentials (grey values in the inset, grey dashed line of the fit) and double exponentials (black values in the inset and red line of the fit. The numbers in parentheses next to the diffusion constant values D_A and D_B indicate the relative weight of each component.

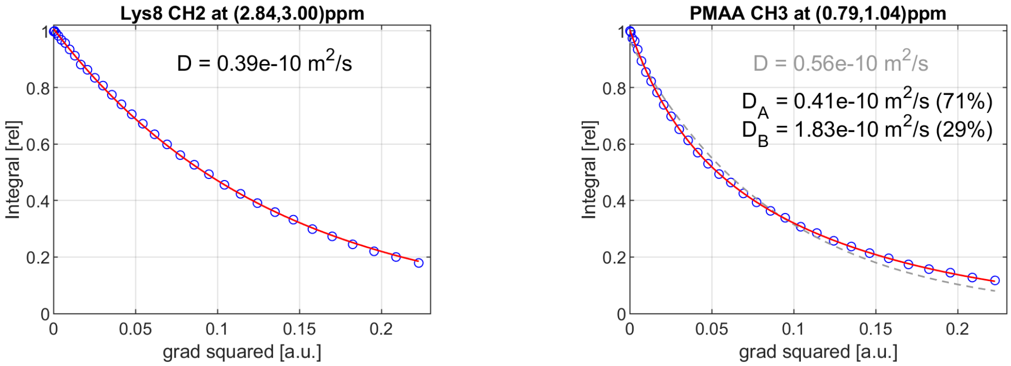

(d) pH = 10.4

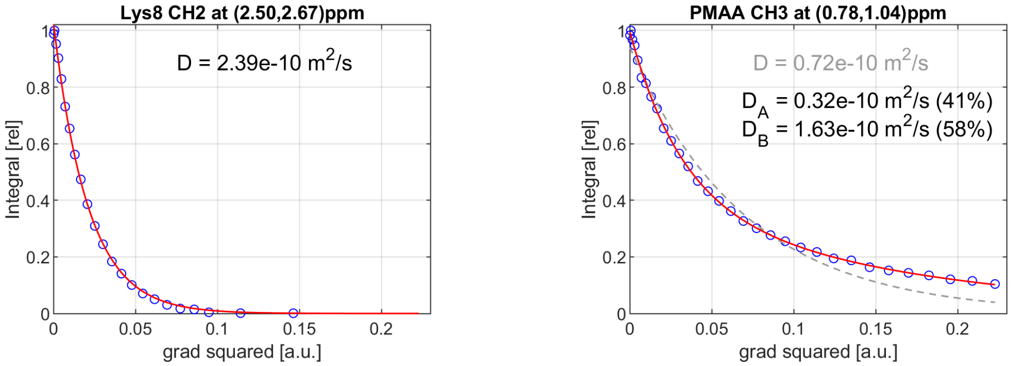

(e) pH = 11.4

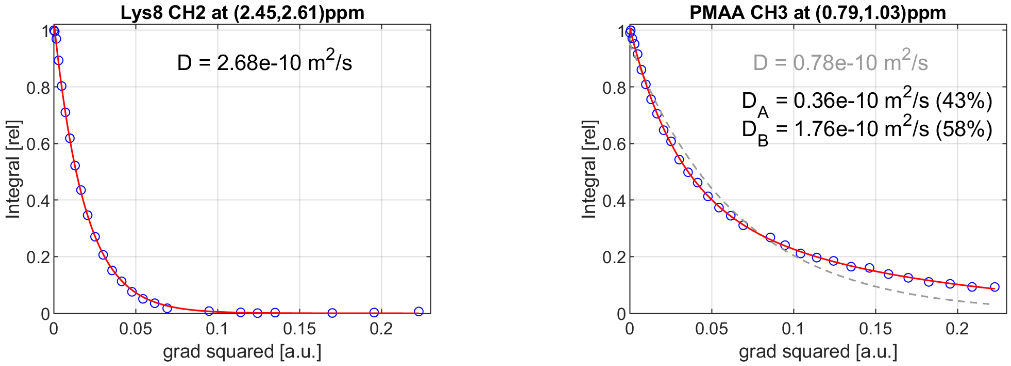

(f) pH = 12.1

Figure S14: (Continued) Evaluation of DOSY spectra of Lys_8 (left) and PMAA (right) in the PMAA + Lys mixture at various pH values. The Lys8 dependencies were fitted using a single exponential. The PMAA dependencies were fitted using both a single exponentials (grey values in the inset, grey dashed line of the fit) and double exponentials (black values in the inset and red line of the fit. The numbers in parentheses next to the diffusion constant values D_A and D_B indicate the relative weight of each component.

2.3.8 NOESY spectra

The red rectangle highlights the region of Lysine CH peak at around 4.5ppm and the terminal CH_2 at around 3ppm in F2 domain, and PMAA CH_3 peaks at around 1ppm in the F1 domain. Crospeaks of varying intensity are observed up to the pH of 10.4. At higher pH values, crospeaks are not present.

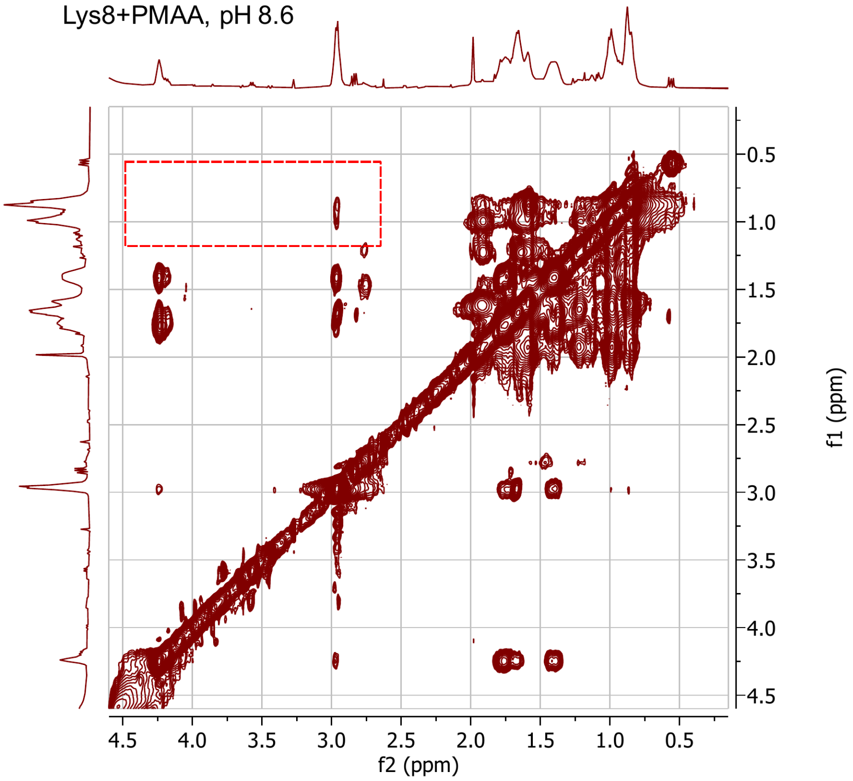

Figure S15: NOESY spectra of PMAA + Lys_8 at various pH values

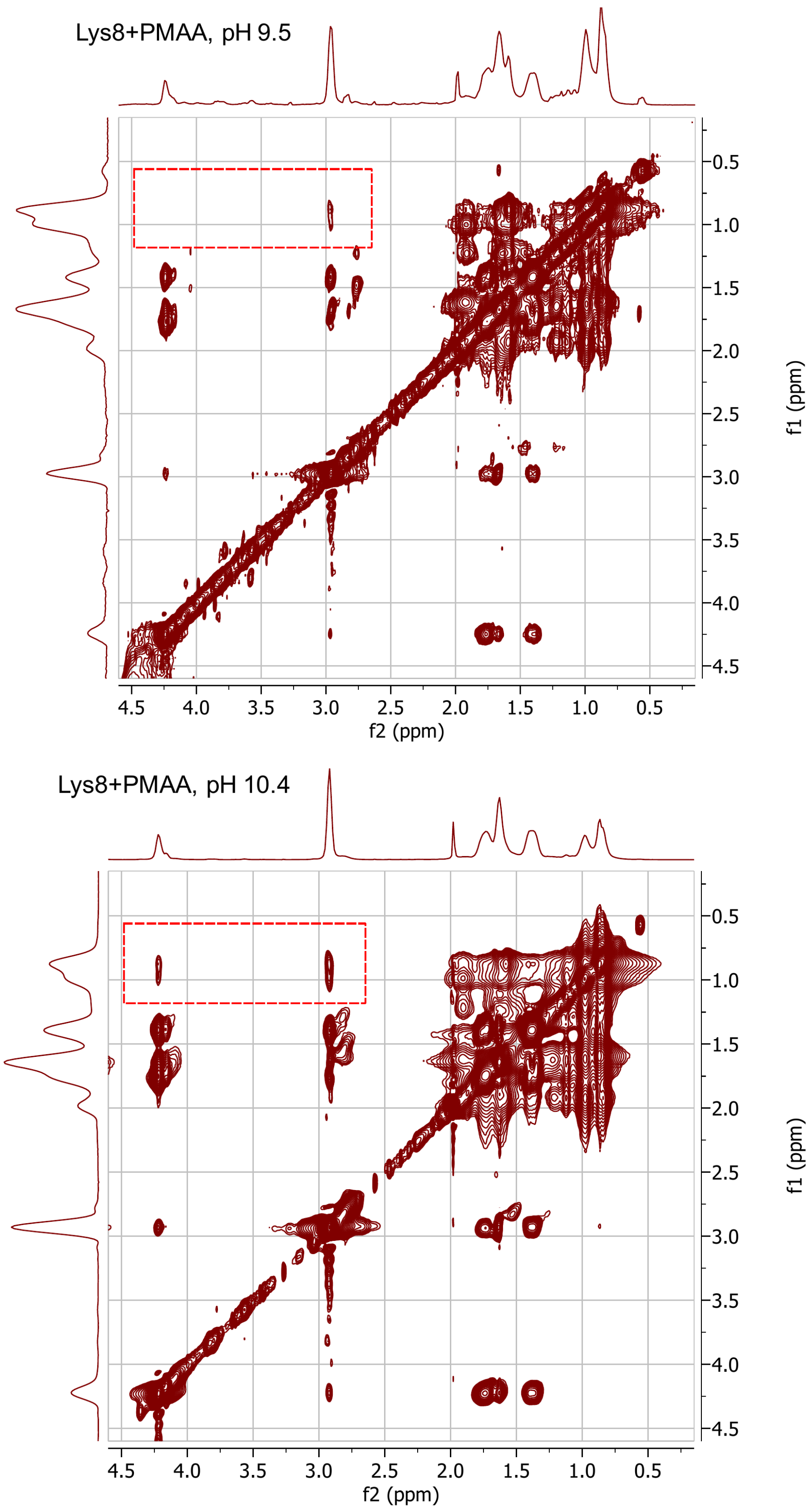

Figure S15: (continued) NOESY spectra of PMAA + Lys_8 at various pH values

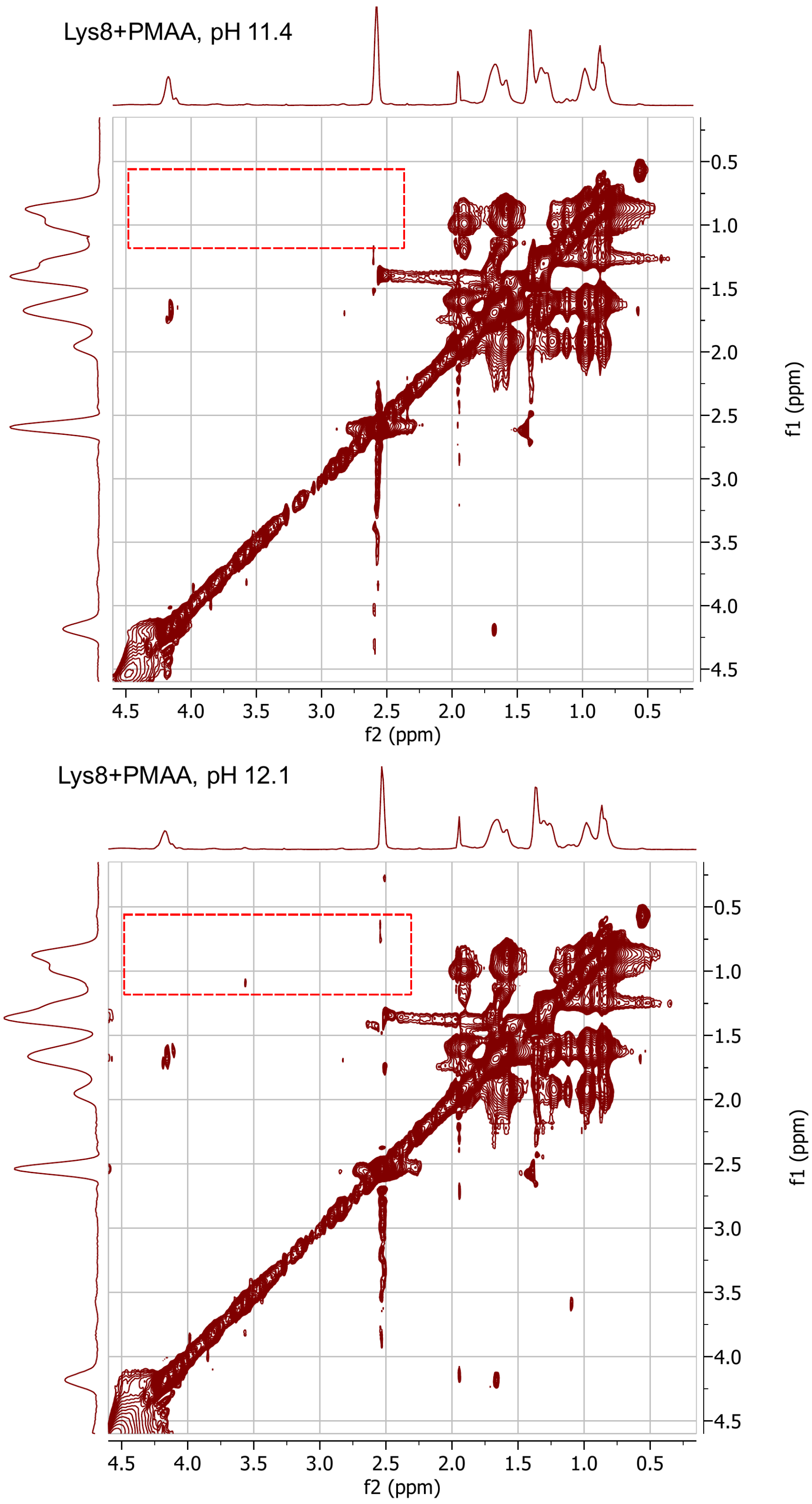

Figure S15: (continued) NOESY spectra of PMAA + Lys_8 at various pH values

References

(S1) Haynes, W. *CRC Handbook of Chemistry and Physics*, 96th ed.; CRC Press: New York, 2015.

(S2) Plavsic, M. B. Configurational Statistics of Poly(acrylic acid). *CROATICA CHEMICA ACTA* **1987**, *60*, 129–137.

(S3) Lunkad, R.; Murmiliuk, A.; Hebbeker, P.; Boublík, M.; Tošner, Z.; Štěpánek, M.; Košovan, P. Quantitative prediction of charge regulation in oligopeptides. *Molecular Systems Design & Engineering* **2021**, *6*, 122–131.

(S4) Hockney, R. W.; Eastwood, J. W. *Computer simulation using particles*; Taylor & Francis, New York, 1988.

(S5) Deserno, M.; Holm, C. How to mesh up Ewald sums. I. A theoretical and numerical comparison of various particle mesh routines. *The Journal of chemical physics* **1998**, *109*, 7678–7693.

(S6) Deserno, M.; Holm, C. How to mesh up Ewald sums. II. An accurate error estimate for the particle–particle–particle-mesh algorithm. *The Journal of chemical physics* **1998**, *109*, 7694–7701.

(S7) Weeber, R.; Grad, J.-N.; Beyer, D.; Blanco, P. M.; Kreissl, P.; Reinauer, A.; Tischler, I.; Košovan, P.; Holm, C. In *Comprehensive Computational Chemistry*, 1st ed.; Yáñez, M., Boyd, R. J., Eds.; Elsevier: Oxford, 2024; pp 578–601.

(S8) Reed, C. E.; Reed, W. F. Monte Carlo study of titration of linear polyelectrolytes. *The Journal of Chemical Physics* **1992**, *96*, 1609–1620.

(S9) Labbez, C.; Jönsson, B. In *Applied Parallel Computing. State of the Art in Scientific Computing*; Kågström, B., Elmroth, E., Dongarra, J., Waśniewski, J., Eds.; Springer: Berlin, Heidelberg, 2007; Vol. 4699; pp 66–72.

(S10) Košovan, P.; Landsgesell, J.; Nová, L.; Uhlík, F.; Beyer, D.; Blanco, P. M.; Staňo, R.; Holm, C. Reply to the 'Comment on "Simulations of ionization equilibria in weak polyelectrolyte solutions and gels"' by J. Landsgesell, L. Nová, O. Rud, F. Uhlík, D. Sean, P. Hebbeker, C. Holm and P. Košovan, Soft Matter, 2019, 15, 1155–1185. *Soft Matter* **2023**, *19*, 3522–3525.

(S11) Janke, W. Statistical Analysis of Simulations: Data Correlations and Error Estimation. *Quantum Simulations of Complex Many-Body Systems: from Theory to Algorithms* **2002**, 423–445.

(S12) Bandura, A. V.; Lvov, S. N. The Ionization Constant of Water over Wide Ranges of Temperature and Density. *Journal of Physical and Chemical Reference Data* **2006**, *35*, 15–30.

(S13) Hwang, T.; Shaka, A. Water Suppression That Works. Excitation Sculpting Using Arbitrary Wave-Forms and Pulsed-Field Gradients. *Journal of Magnetic Resonance, Series A* **1995**, *112*, 275–279.

(S14) Jerschow, A.; Müller, N. Suppression of Convection Artifacts in Stimulated-Echo Diffusion Experiments. Double-Stimulated-Echo Experiments. *Journal of Magnetic Resonance* **1997**, *125*, 372–375.

(S15) Hass, M. A.; Mulder, F. A. Contemporary NMR Studies of Protein Electrostatics. *Annual Review of Biophysics* **2015**, *44*, 53–75, tex.ids: hass2015a.
